# Multicomponent Intervention for Distressed Informal Caregivers of People With Dementia: A Randomized Clinical Trial

**DOI:** 10.1001/jamanetworkopen.2025.0069

**Published:** 2025-03-17

**Authors:** Jojo Yan Yan Kwok, Daphne Sze Ki Cheung, Steven Zarit, Karen Siu-Lan Cheung, Bobo Hi Po Lau, Vivian Weiqun Lou, Sheung-Tak Cheng, Dolores Gallagher-Thompson, Min Qian, Kee-Lee Chou

**Affiliations:** 1 School of Nursing, Li Ka Shing Faculty of Medicine, University of Hong Kong, Hong Kong; 2 Centre on Behavioral Health, Faculty of Social Sciences, University of Hong Kong, Hong Kong; 3 Osher Center on Integrative Health, Brigham and Women’s Hospital, Harvard Medical School, Boston, Massachusetts; 4 School of Nursing, Hong Kong Polytechnic University, Hong Kong; 5 School of Nursing and Midwifery, Centre for Quality and Patient Safety Research, Institute for Health Transformation, Deakin University, Melbourne, Victoria, Australia; 6 Alfred Health, Melbourne, Victoria, Australia; 7 Human Development and Family Studies, Pennsylvania State University, University Park; 8 Sau Po Centre on Ageing, University of Hong Kong, Hong Kong; 9 World Health Organization Collaborating Centre, School of Nursing, Hong Kong Polytechnic University, Hong Kong; 10 Asia-Pacific Institute of Ageing Studies, Lingnan University, Hong Kong; 11 Department of Counselling and Psychology, Hong Kong Shue Yan University, Hong Kong; 12 Department of Health and Physical Education, Education University of Hong Kong, Hong Kong; 13 Department of Psychiatry and Behavioral Sciences, Stanford University School of Medicine, Stanford, California; 14 Mailman School of Public Health, Columbia University, New York, New York; 15 Department of Social Sciences and Policy Studies, Education University of Hong Kong, Hong Kong

## Abstract

**Importance:**

Multicomponent interventions for informal caregivers of people with dementia are urgently needed, but evidence regarding which components are most effective is lacking.

**Objective:**

To apply a multiphase optimization strategy to examine the effects of 5 psychosocial components of an intervention designed to support informal caregivers of people with dementia.

**Design, Setting, and Participants:**

In this assessor-blinded randomized clinical trial with a fractional factorial design, Chinese community-dwelling adults (aged ≥18 years) who were informal family caregivers of people with dementia were screened between July 2 and December 28, 2022, in Hong Kong. Eligible participants with elevated depression or caregiving burden were included and randomized to 1 of 16 experimental conditions. Assessments were conducted at baseline, 6 months, and 12 months. The last 12-month follow-up assessment was conducted on February 26, 2024.

**Interventions:**

The intervention involved a core component (dementia caregiving education) and 5 tested psychosocial components (self-care skills [SC], behavioral problem management [BPM], behavioral activation [BA], mindfulness-based intervention [MBI], and support group [SG]).

**Main Outcomes and Measures:**

Primary outcomes for each tested psychosocial component were physical health (12-item Short-Form Health Survey), caregiver burden (12-item Zarit Burden Interview) and stress (10-item Perceived Stress Scale), psychological well-being (Ryff Psychological Well-Being Scale), anxiety (Hospital Anxiety and Depression Scale–Anxiety Subscale), depressive symptoms (9-item Patient Health Questionnaire), and social support (20-item Medical Outcomes Study Social Support Survey). Multiple linear regression models were used to analyze score changes from baseline to 6 months and from baseline to 12 months for primary and proximal outcomes across the 5 components.

**Results:**

This trial included 250 caregivers (mean [SD] age, 48.9 [13.8] years); most (171 [68.4%]) were female. The MBI component significantly improved multiple caregiver outcomes, with reduced depressive symptoms (β = −2.13 [95% CI, −2.85 to −1.38]; *P* < .001) and increased mindfulness (β = 4.23 [95% CI, 2.27-6.36]; *P* < .001), perceived social support (β = 4.76 [95% CI, 1.28-8.15]; *P* = .007), and active dementia care management (β = 3.70 [95% CI, 1.80-5.66]; *P* < .001) at 12 months. The SG component significantly improved perceived social support (β = 4.63 [95% CI, 1.32-7.85]; *P* = .006) at 12 months. BPM had mixed effects; that is, it initially increased caregiver anxiety (β = 1.43 [95% CI, 0.43-2.42]; *P* = .005) and self-care risk (β = −1.12 [95% CI, −1.82 to −0.43]; *P* = .002) at 6 months but improved dementia care strategies in terms of encouragement (β = 2.49 [95% CI, 0.74-4.22]; *P* = .005), active management (β = 5.99 [95% CI, 4.12-7.84]; *P* < .001), and psychological well-being (β = 3.52 [95% CI, 0.92-6.08]; *P* = .008) at 12 months. Interaction effects were observed, with the MBI component enhancing the benefits of SC (β = −1.70 [95% CI, −3.05 to −0.35]; *P* = .01) and BPM (β = −1.40 [95% CI, −2.76 to −0.05]; *P* = .04) on depression; meanwhile, the MBI and SG components synergistically improved perceived social support (β = 7.58 [95% CI, 0.90-14.26]; *P* = .03).

**Conclusions and Relevance:**

In this clinical trial of informal caregivers of people with dementia, synergistic interaction effects were noted for MBI, which enhanced the benefits of SC and BPM on depression. The combination of the MBI and SG components also synergistically improved social support. Integrating MBI with SC, SG, or BPM components was an effective multicomponent approach to support caregivers in this study, although ongoing support was needed to mitigate potential short-term risks. Further research is required to validate the efficacy of this optimized intervention package.

**Trial Registration:**

Chinese Clinical Trial Registry Identifier: ChiCTR2300071235

## Key Points

**Question:**

In a multimodal support intervention for distressed informal caregivers of people with dementia, what psychosocial components (self-care, behavioral problem management, behavioral activation, mindfulness, or support group) are most effective?

**Findings:**

In this randomized clinical trial of 250 caregivers with depressive symptoms or caregiving burden, synergistic interaction effects were noted for mindfulness, thereby enhancing the benefits of self-care and behavioral problem management on depression. The mindfulness and support group components synergistically improved perceived social support.

**Meaning:**

These findings suggest that integrating mindfulness with support group or behavioral management components could be an efficient and effective multicomponent approach to improve psychosocial well-being among caregivers of people with dementia.

## Teaser

This randomized clinical trial uses a multiphase optimization strategy to examine the effectiveness of the psychosocial components of an intervention designed to support distressed informal caregivers of Chinese community-dwelling adults with dementia in Hong Kong.

## Introduction

Dementia is a growing global health crisis that affects more than 55 million people worldwide. With the aging of the population, this number of older people is expected to reach approximately 78 million by 2030 and 139 million by 2050.^1^ The global financial burden is estimated at $1.3 trillion, potentially reaching $2.8 trillion by 2030. Informal caregivers play an important role in long-term care of people with dementia but face substantial negative consequences, including high stress, severe burden, anxiety, depression, and increased risk of cardiovascular disease.^2^ These consequences affect the well-being of caregivers and care recipients, while also increasing health care and long-term care costs due to early institutionalization. Effective interventions are urgently needed to mitigate the adverse effects of dementia caregiving.

In a 2020 meta-analysis of 131 randomized clinical trials (RCTs), investigators found that multicomponent interventions have the most notable effect on reducing caregiver burden and stress and enhancing well-being.^3^ However, it remains unclear which specific components or combinations are most effective. Typical multicomponent interventions, similar to Resources for Enhancing Alzheimer’s Caregiver Health (REACH),^4,5,6^ include psychoeducation, self-care skills (SC), behavioral problem management (BPM), behavioral activation (BA), mindfulness-based intervention (MBI), and social support group (SG) components. Clinical trials have compared various psychosocial interventions for dementia caregiving and produced mixed results.^7,8,9,10^ Components and their interaction that contribute to positive effects on dementia caregivers remain unknown. Essential effective components should be identified to efficiently deliver these interventions to dementia caregivers. However, to our knowledge, no study has yet provided evidence on the optimal components or combinations for family dementia caregivers.

The multiphase optimization strategy (MOST) framework, developed by Collins et al,^11^ is a systematic approach designed to identify the most effective components of multicomponent interventions. The framework comprises 3 distinct phases—preparation, optimization, and evaluation—and its usefulness has been demonstrated across several domains, such as smoking cessation, physical activity promotion, obesity reduction, alcohol use reduction, unsafe sex prevention, and education intervention.^12^ However, application of the MOST framework within gerontology or caregiving contexts remains unexplored. This study primarily focused on the preparation and optimization stages of the MOST framework.^13^ The preparatory phase involved a comprehensive review of existing literature, consultations with experts, and preliminary studies to determine potential intervention components based on theoretical or empirical justification. This approach facilitates an independent evaluation of each component. The optimization phase, on the other hand, focused on the empirical testing of the identified components through randomized experiments to assess their individual and interactive effects on desired outcomes.

The overarching goal of this optimization trial was to identify the most effective combination of intervention components, recognizing the possible presence of more than 1 effective combination, and to develop a matrix of intervention component combinations tailored to different distress profiles. This study aimed to examine the main effects and 2-way interactions^11^ of the 5 individual intervention components on their corresponding primary outcomes (SC: physical health; BPM: caregiving burden and stress; BA: psychological well-being; MBI: anxiety and depression; and SG: perceived social support) and proximal outcomes (SC: self-care risks; BPM: dementia care strategies; BA: engagement in pleasurable activities; MBI: trait mindfulness; and SG: satisfaction with support group) among dementia caregivers with moderate caregiving burden or depressive symptoms.

## Methods

The trial protocol was approved by the Institutional Review Board of the Education University of Hong Kong, Human Research Ethics Committee. The full protocol, including detailed descriptions of the intervention and statistical analysis plan, has been published previously.^13^ The formal trial protocols can be found in Supplement 1. Participants provided written informed consent. The study followed the Consolidated Standards of Reporting Trials (CONSORT) reporting guideline and its extension to nonpharmacologic interventions.

### Study Design

We conducted this multicenter, eHealth-assisted, remotely delivered component selection, assessor-blinded RCT with a fractional factorial design with 16 experimental conditions (eTable 1 in Supplement 2). We implemented the aforementioned MOST phases to evaluate the effects of 5 individual psychosocial intervention components (SC, BPM, BA, MBI, and SG) and their 2-way interactions among family dementia caregivers at 6 months (immediate effect) and 12 months (long-term effect).^11^ By adhering to these phases, we aimed to develop interventions that are not only effective but also practical and scalable for clinical application.^14^ Psychoeducation was designated as the core component in this trial, given its extensive study and routine use in supporting dementia caregivers. Participants were randomly assigned to the presence or absence of each of the 5 experimental intervention components.^15^ This methodology allowed us to examine the effects of these components and their combinations, a departure from a full factorial design that would have necessitated 32 experimental conditions. Usual care was not regarded as the control group because each experimental condition could serve as a control condition under different circumstances, enabling the testing of individual component effects. This article reports detailed analyses of the corresponding primary outcomes, proximal outcomes, and the 2-way interaction effects of all the tested intervention components.

### Participants

We recruited participants through convenience sampling from psychiatric clinics, psychogeriatric clinics, and nongovernmental organizations (NGOs) providing support services to older adults, individuals with dementia, or both between July 2, 2022, and December 28, 2022. We also advertised the study through newspapers, radio, and social media platforms to reach additional participants.

The study had several inclusion criteria. To ensure sample homogeneity, participants were required to (1) be a Hong Kong Chinese citizen aged 18 years or older; (2) be a spouse, adult child, or child-in-law of a care recipient; (3) have no cognitive impairment based on the 5-Minute Hong Kong version of the Montreal Cognitive Assessment with an age and education-specific cutoff score^16^; (4) have served as the primary informal caregiver for a person with a clinical diagnosis of dementia for at least 1 year, having dedicated a minimum of 20 hours per week to caregiving and assisting with activities of daily living (ADLs) and instrumental ADLs; and (5) be a caregiver experiencing a certain level of depression (Patient Health Questionnaire-9 [PHQ-9] score >9) or caregiving burden (Zarit Burden Interview Scale [ZBI] score >18).^17,18^ As a token of appreciation, participants received a financial incentive in the form of a US $51 supermarket voucher upon completion of the intervention and all assessments.

### Screening, Baseline Testing, Randomization, and Blinding

On the informed consent form, participants were only informed that the study aimed to support dementia caregiving, without revealing specific intervention details or claiming component superiority. The number of intervention groups was also not disclosed.

After baseline assessment, eligible participants were randomly assigned to 1 of 16 experimental conditions, with a 1:1 allocation to the presence or absence of each intervention component. The randomization sequence was generated by an independent research assistant using an online random number generator. An independent researcher who was not involved in assessment informed participants of their assigned group, and the allocation list was concealed from the other research team members and participants until assignment.

eTable 1 in Supplement 2 lists the 16 experimental conditions in the fractional factorial design, with the number of participants assigned to each. Each condition included the psychoeducation core component plus a different combination of the 5 psychosocial intervention components.

Outcome assessors were blinded to participant group assignment. Given the behavioral nature of the interventions, blinding was not possible for participants. Primary and proximal outcome measures were self-reported by participants, and the statistician analyzing the dataset was kept blinded until the results were finalized.

### Fidelity Monitoring

The intervention was delivered via telephone and videoconferencing software (Zoom) by trained research assistants. Interventionists received 10 hours of intensive training, which included reading materials, structured role-play, and return demonstration opportunities for each component. To ensure adherence to the intervention protocols, interventionists submitted taped sessions, and the research team reviewed the initial session plus at least 20% of the recordings throughout the study to provide feedback to the interventionists. Furthermore, an intervention fidelity checklist was used in each session to record the number and duration of each intervention session as well as caregivers’ enactment in 4 aspects: data collection, home assignments, use of notebooks, and use of written prescriptions.^19^ The research team closely monitored the intervention implementation through weekly supervision meetings, monthly conference calls, and review and feedback of taped intervention sessions and fidelity checklists, involving all interventionists. A satisfaction questionnaire was administered to all participants after the completion of the intervention to better understand their satisfaction with specific components and the quality of services.^20^

### Intervention

The intervention included psychoeducation as the core component plus the 5 experimental components (SC, BPM, BA, MBI, and SG). All participants received the psychoeducation core component within 2 weeks after consent, and baseline assessments were obtained. They were randomized to receive either the presence or absence of each experimental component.

Consistent with pragmatic research principles,^21^ all components, duration, and delivery systems were designed for community application, with an emphasis on brevity while covering essential materials. As a result, there was some variation in the amount of time allocated to each of the 5 components. Except for experimental component 5, which involved a support group intervention, all other components of the intervention were individually based. On average, each component took 3 weeks to complete. Content for the psychoeducation, SC, BPM, and BA components was adapted from the extensively studied REACH intervention.^4,5,6^

#### Psychoeducation Core Component

The psychoeducation core component was provided to all participants and consisted of one 30-minute videoconferencing group session and two 30-minute individual telephone or videoconferencing sessions, which were delivered by well-trained research assistants. Information was provided via presentation slides, which were also available in audio and video formats. The content covered general dementia knowledge, communication skills for interacting with people with dementia, and common caregiving problems and seeking help. The first 2 sessions included safety walkthroughs via videoconference, during which participants received advice about ensuring home safety through modifications.

#### Experimental Psychosocial Components

##### Self-Care Skills

For the SC experimental component, participants received 1 hour of online training materials in the form of slides, audio, and video, as well as two 30-minute follow-up telephone calls delivered by research assistants. The content covered the importance of maintaining good health and continuing healthy behavior for both caregivers and care recipients, along with the use of health passports to track health conditions and medical appointments.

##### Behavioral Problem Management

For the BPM experimental component, participants received one 1-hour and two 30-minute telephone calls from research assistants. Participants were introduced to the antecedent-behavior-consequence model to manage the behavioral symptoms exhibited by their care recipients. Participants were trained in observing, recording, and developing plans to address problem behaviors, and they were encouraged to complete home practices regularly.

##### Behavioral Activation

For the BA experimental component, participants received five 1-hour telephone calls from research assistants. They were encouraged to schedule pleasant activities into their daily routine, following a telephone-based protocol used in previous studies of Chinese caregivers.^5,22^ Specifically, caregivers were taught about the principles of BA and participated in sessions on activity monitoring, activity scheduling, reinforcing or modifying a pleasant event, and activity rescheduling based on changes after modification.

##### Mindfulness-Based Intervention

For the MBI experimental component, participants received seven 2-hour videoconferencing group sessions delivered by a certified mindfulness teacher. The content was modified from mindfulness-based cognitive therapy and included developing an understanding of mindfulness, cultivating awareness of thoughts and emotions, practicing mindfulness exercises such as body scanning and breathing techniques, introducing mindfulness stretching, exploring acceptance of negative emotions, reevaluating thoughts and reality, and fostering self-compassion. Each session focused on a specific theme and relevant mindfulness activities (eg, in the first session, “Waking Up from Automatic Pilot,” participants practiced body scanning and raisin-eating meditation to enhance mindfulness and awareness of the present moment). The component concluded with a session reviewing the content and encouraging participants to continue daily mindfulness practices.

##### Social Support Group

For the SG experimental component, participants received six 1-hour videoconferencing group sessions covering 6 major themes^23^: (1) introduction to dementia caregiving and establishing a mutual support group, (2) enhancing home care skills and interpersonal relationships, (3) awareness of caregivers’ mental health, (4) using community resources, (5) sharing experiences on behavioral symptom management, and (6) concluding and reviewing the program. These sessions involved sharing information, facilitating group discussion, providing psychological support, and engaging participants in problem-solving exercises. An experienced social worker acted as the facilitator, while 2 peer leaders were elected by the group members.

### Outcome Measures

In the MOST preparation phase, we developed a conceptual model (eFigure 1 in Supplement 2) based on the theoretical framework model for the stress–health process of informal caregivers of people with dementia.^24^ eFigure 2 in Supplement 2 illustrates how each component affects its proximal outcome, which in turn affects the primary outcome.

#### Primary Outcome Measures

##### Physical Health

For the SC component, physical health status was measured using the physical health component of the validated 12-item Short-Form Health Survey (SF-12).^25^ Higher scores indicated a better health condition.

##### Caregiving Burden and Stress

For the BPM component, caregiver burden and general stress were measured with the 12-item ZBI^26^ and the 10-item Perceived Stress Scale (PSS),^27^ respectively. Both used a 5-point Likert scale, with higher scores indicating greater caregiver burden and higher levels of stress.

##### Psychological Well-Being

For the BA component, psychological well-being was measured with the 4 subscales of the Ryff Psychological Well-Being Scale (PWB) (ie, self-acceptance, positive relations to others, purpose of life, and personal growth, with 4 items in each subscale).^28^ Participants were asked to rate the items on a 6-point Likert scale, with higher scores indicating better psychological well-being.

##### Anxiety and Depression

For the MBI component, anxiety and depressive symptoms were measured using the 7-item Chinese version of the Hospital Anxiety and Depression Scale–Anxiety Subscale (HADS-A)^29^ and the 9-item Chinese version of the PHQ-9,^17^ respectively. Both used a 4-point Likert scale, with higher scores indicating more anxiety and depressive symptoms.

##### Perceived Social Support

For the SG component, perceived adequacy of functional social support was measured using the 20-item Medical Outcomes Study Social Support Survey (MOS-SSS).^30,31^ Participants rated their responses on a 5-point Likert scale, with higher scores indicating a higher level of social support.

#### Proximal Outcome Measures

##### Self-Care Risks

For the SC component, self-care was measured using the 14-item Self-Care subscale of the Risk Appraisal Measure (RAM-SC).^6^ This subscale captured the unique risk profile of caregivers in terms of self-care. High scores indicated a low risk for caregivers.

##### Dementia Care Strategies

For the BPM component, caregiving strategies were measured using the 34-item Dementia Management Strategies Scale (DMSS).^4,6^ The DMSS used a 5-point Likert scale and included 3 domains: criticism, encouragement, and active management.

##### Engagement in Pleasurable Activities

For the BA component, engagement in pleasurable activities was assessed. Patients reported the number of meaningful or joyful events they had experienced over the past 2 weeks.

##### Trait Mindfulness

For the MBI component, mindfulness was measured using the 20-item version of the Five Facet Mindfulness Questionnaire (FFMQ).^28^ The FFMQ used a 5-point Likert scale and assessed 5 facets of mindfulness: observing (4 items), describing (4 items), acting with awareness (4 items), nonjudging of inner experience (4 items), and nonreactivity to inner experience (4 items). Higher scores indicated a higher level of mindfulness.

##### Support Group Satisfaction

For the SG component, satisfaction was assessed using a 7-item scale administered after the intervention. Participants reported their satisfaction with specific components and the quality of service.^20^

### Sample Size

The fractional factorial MOST design relied on the smallest clinically important difference between the presence and absence of a component; this was used to determine the required sample size for detecting main effects, rather than the number of components evaluated. Previous research suggested that an effect size of 0.60 (Cohen *d*) at 6 months is sufficient for a quasi-experimental pre-post treatment without a control group to determine the sample size.^6^ However, we adopted a more conservative effect size estimate of 0.40 for this study, with 12-month follow-up as the primary end point. Assuming an attrition rate of 20%, a sample size of 250 participants would provide 80% power to detect a main effect or interaction effect size of 0.40 at an α level of .05 using a 2-tailed hypothesis test. To ensure equal allocation to 16 experimental conditions, recruiting 256 participants (16 participants per each experimental condition) was deemed necessary. The sample size calculation was performed using G*Power, version 3.1 (G*Power Team).

### Statistical Analysis

The intention-to-treat (ITT) principle was adopted for data analysis. Missing data were handled through multiple imputation using demographic information. For participants with follow-up data, separate regression models were computed to regress the scale variables with missing values on demographic variables (age, sex, and educational attainment). Applying the demographic information and the coefficients from these regression models, estimated values of the scale variables were determined for participants with missing data. Separate multiple linear regression models were computed to analyze the change in scores from baseline to 6 months and from baseline to 12 months for primary and proximal outcomes for the 5 components, respectively. A total of 7 regression models were used to analyze the primary outcomes, whereas an additional 7 regression models were used for the proximal outcomes. The analysis was adjusted for a set of covariates, including age, sex, educational attainment, number of components the participants had, number of years of caregiving, and whether the caregivers were the sole caregivers. Although randomization minimizes confounding issues, we adjusted for these variables to enhance precision and account for potential baseline imbalances. This strategy improved statistical efficiency and strengthened our ability to detect true intervention effects by reducing unexplained variance. The selection of adjustment variables was based on their theoretical importance and was prespecified in our statistical analysis plan.^13^ In the present study, we tested 5 intervention components, each associated with 1 to 2 hypothesized primary outcomes. To account for multiplicity, a 1% level of significance (*P* < .01) was assumed for primary and proximal analyses, whereas a 5% level of significance (*P* < .05) was assumed for exploratory analyses regarding 2-way interaction effects of intervention components. All significance tests were 2 sided. SPSS, version 26.0 (IBM SPSS Statistics), was used for all analyses.

## Results

**Figure 1.**
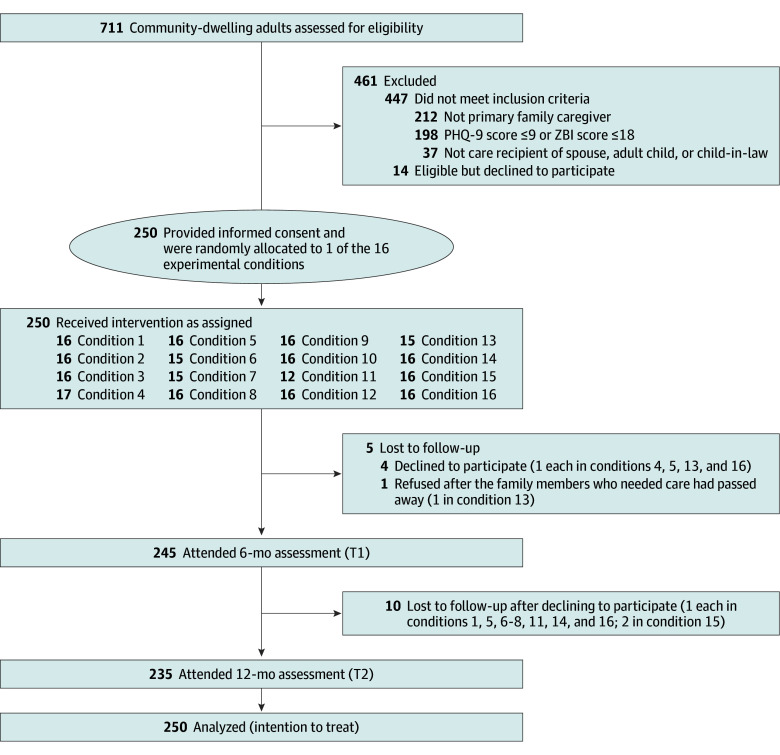
CONSORT Flow Diagram Condition 1: psychoeducation, self-care skill training, behavioral symptom management, behavioral activation, mindfulness-based intervention, social support group. Condition 2: psychoeducation, self-care skill training, behavioral symptom management, behavioral activation. Condition 3: psychoeducation, self-care skill training, behavioral symptom management, behavioral activation. Condition 4: psychoeducation, self-care skill training, behavioral symptom management, social support group. Condition 5: psychoeducation, self-care skill training, behavioral activation, mindfulness-based intervention. Condition 6: psychoeducation, self-care skill training, behavioral activation, social support group. Condition 7: psychoeducation, self-care skill training, behavioral activation, social support group. Condition 8: psychoeducation, self-care skill training. Condition 9: psychoeducation, behavioral symptom management, behavioral activation, mindfulness-based intervention. Condition 10: psychoeducation, behavioral symptom management, behavioral activation. Condition 11: psychoeducation, behavioral symptom management, behavioral activation, social support group. Condition 12: psychoeducation, behavioral symptom management. Condition 13: psychoeducation, behavioral activation, mindfulness-based intervention, social support group. Condition 14: psychoeducation, behavioral activation. Condition 15: psychoeducation, behavioral activation. Condition 16: psychoeducation, social support group. PHQ-9 indicates Patient Health Questionnaire-9; ZBI, Zarit Burden Interview Scale.

We screened 711 caregivers of people with dementia from July 2 to December 28, 2022. The last 12-month follow-up assessment was conducted on February 26, 2024. Among the 264 eligible individuals, 250 consented to participate (consent rate, 94.7%) and were randomized to 1 of the 16 experimental conditions. Figure 1 illustrates participant flow through the study. Table 1 summarizes the baseline demographic and caregiving characteristics of participants and their care recipients. The mean age (SD) was 48.9 (13.8) years for caregivers and 76.7 (8.9) years for care recipients. Women comprised the majority of both groups, with 171 female (68.4%) vs 79 male (31.6%) caregivers and 154 female (61.6%) vs 96 male (38.4%) care recipients. Most caregivers had educational attainment of secondary education or less (156 [62.4%]), were employed (156 [62.4%]), and lived with the care recipient or recipients (201 [80.4%]). Almost 90% of caregivers were either adult children (190 [76.0%]) or children-in-law (28 [11.2%]) of the care recipients, and they spent a mean (SD) of 59.5 (46.0) hours per week on caregiving. A total of 187 care recipients (74.8%) had a comorbidity, and 48 (19.2%) had a disability. No statistically significant differences in baseline characteristics of caregivers and their care recipients were observed between the presence and absence groups for each of the 5 intervention components.

**Table 1.** Baseline Characteristics of Family Caregivers and Care Recipients With Dementia by Each Component^a^

Characteristic	All caregivers (N = 250)	Psychosocial component									
		Self-care skills		Behavioral problem management		Behavioral activation		Mindfulness-based intervention		Social support group	
		Presence (n = 127)	Absence (n = 123)	Presence (n = 125)	Absence (n = 125)	Presence (n = 126)	Absence (n = 124)	Presence (n = 122)	Absence (n = 128)	Presence (n = 122)	Absence (n = 128)
**Caregivers**												
Age, mean (SD), y	48.9 (13.8)	51.7 (14.0)	46.0 (13.0)	50.5 (13.0)	47.2 (14.3)	50.2 (13.7)	47.5 (13.8)	53.0 (14.1)	44.9 (12.2)	49.0 (14.3)	48.8 (13.3)
Sex											
Male	79 (31.6)	39 (30.7)	40 (32.5)	45 (36.0)	34 (27.2)	31 (24.6)	48 (38.7)	35 (28.7)	44 (34.4)	39 (32.0)	40 (31.3)
Female	171 (68.4)	88 (69.3)	83 (67.5)	80 (64.0)	91 (72.8)	95 (75.4)	76 (61.3)	87 (71.3)	84 (65.6)	83 (68.0)	88 (68.8)
Education level											
Secondary or less	156 (62.4)	87 (68.5)	69 (56.1)	82 (65.6)	74 (59.2)	82 (65.1)	74 (59.7)	86 (70.5)	70 (54.7)	71 (58.2)	85 (66.4)
Tertiary or greater	94 (37.6)	40 (31.5)	54 (43.9)	43 (34.4)	51 (40.8)	44 (34.9)	50 (40.3)	36 (29.5)	58 (45.3)	51 (41.8)	43 (33.6)
Employment status											
Unemployed	94 (37.6)	55 (43.3)	39 (31.7)	53 (42.4)	41 (32.8)	58 (46.0)	36 (29.0)	59 (48.4)	35 (27.3)	39 (32.0)	55 (43.0)
Employed	156 (62.4)	72 (56.7)	84 (68.3)	72 (57.6)	84 (67.2)	68 (54.0)	88 (71.0)	63 (51.6)	93 (72.7)	83 (68.0)	73 (57.0)
Relationship with care recipient											
Spouse	32 (12.8)	21 (16.5)	11 (8.9)	19 (15.2)	13 (10.4)	21(16.7)	11 (8.9)	25 (20.5)	7 (5.5)	20 (16.4)	12 (9.4)
Adult child	190 (76.0)	90 (70.9)	100 (81.3)	90 (72.0)	100 (80.0)	91 (72.2)	99 (79.8)	87 (71.3)	103 (80.5)	94 (77.0)	96 (75.0)
Child-in-law	28 (11.2)	16 (12.6)	12 (9.8)	16 (12.8)	12 (9.6)	14 (11.1)	14 (11.3)	10 (8.2)	18 (14.1)	8 (6.6)	20 (15.6)
Living with care recipient	201 (80.4)	99 (78.0)	102 (82.9)	102 (81.6)	99 (79.2)	104 (82.5)	97 (78.2)	97 (79.5)	104 (81.3)	103 (84.4)	98 (76.6)
Sole caregiver	89 (35.6)	49 (38.6)	40 (32.5)	44 (35.2)	45 (36.0)	54 (42.9)	35 (28.2)	51 (41.8)	38 (29.7)	45 (36.9)	44 (34.4)
Duration of caregiving, y	4.3 (3.5)	4.3 (3.7)	4.2 (3.3)	4.3 (2.9)	4.3 (4.1)	4.4 (3.9)	4.1 (3.2)	4.6 (3.8)	3.9 (3.2)	4.4 (4.0)	4.2 (3.1)
Time spent on caregiving, mean (SD), h/wk	59.5 (46.0)	59.5 (48.9)	59.5 (43.1)	60.2 (48.7)	58.8 (43.3)	68.7 (50.7)	50.1 (38.7)	65.1 (49.8)	54.2 (41.6)	66.0 (51.4)	53.3 (39.4)
**Care recipients**												
Age, mean (SD), y	76.7 (8.9)	77.5 (8.8)	76.0 (9.0)	77.4 (8.4)	76.0 (9.4)	77.4 (9.2)	76.0 (8.6)	78.2 (8.6)	75.4 (9.1)	76.5 (9.3)	77.0 (8.6)
Sex											
Male	96 (38.4)	53 (41.7)	43 (35.0)	50 (40.0)	46 (36.8)	57 (45.2)	39 (31.5)	41 (33.6)	55 (43.0)	55 (45.1)	41 (32.0)
Female	154 (61.6)	74 (58.3)	80 (65.0)	75 (60.0)	79 (63.2)	69 (54.8)	85 (68.5)	81 (66.4)	73 (57.0)	67 (54.9)	87 (68.0)
Presence of comorbidity	187 (74.8)	95 (74.8)	92 (74.8)	104 (83.2)	83 (66.4)	93 (73.8)	94 (75.8)	96 (78.7)	91 (71.1)	89 (73.0)	98 (76.6)
Disability	48 (19.2)	30 (23.6)	18 (14.6)	27 (21.6)	21 (16.8)	21 (16.7)	27 (21.8)	24 (19.7)	24 (18.8)	14 (11.5)	34 (26.6)

^a^ Values are presented as No. (%) of caregivers or care recipients.

Of the 250 caregivers, 245 (98.0%) completed the 6-month follow-up assessment and 235 (94.0%) completed the 12-month follow-up assessment. The attrition rates for the 6-month and 12-month follow-up assessments were 2.0% and 6.0%, respectively. For implementation fidelity, all participants attended the assigned sessions with set durations, completed the home assignments, read, and used the provided notebooks and written prescriptions as presented in the materials. Participants reported equally high satisfaction scores across the 5 experimental components (eTable 2 in Supplement 2). One care recipient of the participant in condition 13 (which entailed a combination of dementia caregiving education and BA, MBI, and SG components) died during the study period, but the death was determined to be unrelated to the trial intervention because the care recipient had unrestricted access to usual health care and was not subjected to any lifestyle restrictions. No other adverse events were reported during the trial.

### Main Effects of Intervention Components

#### Hypothesized Primary Outcomes

Following ITT analyses, Table 2 presents the main effects of the intervention components on the corresponding primary outcomes over a 12-month period. For the hypothesized primary outcomes, the MBI component significantly reduced depressive symptoms at 12 months (PHQ-9: β = −2.13 [95% CI, −2.85 to −1.38]; *P* < .001), and the SG component significantly improved perceived social support at 12 months (MOS-SSS: β = 4.63 [95% CI, 1.32-7.85]; *P* = .006). Beyond the hypothesized primary outcomes, the BPM component significantly improved physical health (SF-12: β = 2.44 [95% CI, 0.72-4.15]; *P* = .006) and was associated with increased anxiety at 6 months (HADS-A: β = 1.43 [95% CI, 0.43-2.42]; *P* = .005) and improved psychological well-being at 12 months (PWB: β = 3.52 [95% CI, 0.92-6.08]; *P* = .008), and the MBI component significantly improved perceived social support at 12 months (MOS-SSS: β = 4.76 [95% CI, 1.28-8.15]; *P* = .007).

**Table 2.** Adjusted Regression Analyses for Change in Primary Outcomes of 5 Components Over 12 Months

Outcome (assessment tool)^a^	Psychosocial component									
	Self-care skills		Behavioral problem management		Behavioral activation		Mindfulness-based intervention		Social support group	
	β (95% CI)	*P* value^b^	β (95% CI)	*P* value	β (95% CI)	*P* value	β (95% CI)	*P* value	β (95% CI)	*P* value
**Hypothesized primary outcome**										
Physical health (SF-12^c^)										
0-6 mo, Δ	1.48 (−0.25 to 3.21)	.09	2.44 (0.72-4.15)	.006	−0.65 (−2.38 to 1.08)	.46	1.15 (−0.63 to 2.93)	.21	0.49 (−1.20 to 2.18)	.57
0-12 mo, Δ	0.36 (−1.14 to 1.85)	.64	0.58 (−0.89 to 2.07)	.44	−1.51 (−3.01 to −0.02)	.05	1.17 (−0.37 to 2.70)	.14	−0.06 (−1.52 to 1.40)	.94
Caregiving burden and stress										
Caregiving burden (ZBI^d^)										
0-6 mo, Δ	0.90 (−1.01 to 2.81)	.35	1.65 (−0.24 to 3.54)	.09	−0.68 (−2.60 to 1.23)	.49	2.48 (0.52-4.45)	.014	0.45 (−1.42 to 2.32)	.64
0-12 mo, Δ	1.07 (−0.81 to 2.90)	.26	−0.21 (−2.07 to 1.61)	.83	−1.56 (−3.40 to 0.32)	.10	1.42 (−0.44 to 3.38)	.15	1.18 (−0.59 to 3.04)	.20
General stress (PSS^e^)										
0-6 mo, Δ	−0.40 (−1.75 to 0.95)	.56	0.61 (−0.73 to 1.94)	.37	−0.39 (−1.74 to 0.97)	.58	1.26 (−0.13 to 2.65)	.08	0.44 (−0.88 to 1.76)	.52
0-12 mo, Δ	0.70 (−0.57 to 1.99)	.28	−0.05 (−1.31 to 1.22)	.94	−1.32 (−2.61 to −0.05)	.04	0.66 (−0.68 to 1.95)	.33	−0.53 (−1.79 to 0.71)	.41
Psychological well-being (PWB^f^)										
0-6 mo, Δ	0.48 (−1.94 to 2.90)	.70	2.44 (0.05-4.84)	.05	−1.99 (−4.41 to 0.43)	.11	0.38 (−2.11 to 2.86)	.77	0.50 (−1.86 to 2.87)	.67
0-12 mo, Δ	0.05 (−2.58 to 2.64)	.97	3.52 (0.92-6.08)	.008	1.49 (−1.11 to 4.12)	.27	3.00 (0.35-5.72)	.03	0.24 (−2.28 to 2.82)	.85
Anxiety and depression										
Anxiety symptoms (HADS-A^g^)										
0-6 mo, Δ	−0.43 (−1.43 to 0.57)	.40	1.43 (0.43-2.42)	.005	−0.53 (−1.54 to 0.47)	.30	−0.85 (−1.88 to 0.18)	.11	0.38 (−0.59 to 1.36)	.44
0-12 mo, Δ	0.00 (−0.92 to 0.96)	>.99	0.56 (−0.34 to 1.52)	.23	−0.46 (−1.42 to 0.46)	.34	−1.07 (−2.09 to −0.16)	.03	−0.06 (−1.02 to 0.82)	.90
Depressive symptoms (PHQ-9^h^)										
0-6 mo, Δ	0.25 (−0.13 to 0.64)	.20	−0.05 (−0.43 to 0.33)	.80	0.19 (−0.19 to 0.58)	.32	0.31 (−0.08 to 0.71)	.12	0.28 (−0.09 to 0.66)	.14
0-12 mo, Δ	−0.23 (−0.95 to 0.47)	.52	−0.38 (−1.09 to 0.32)	.29	−0.29 (−1.00 to 0.43)	.43	−2.13 (−2.85 to −1.38)	<.001	0.35 (−0.34 to 1.06)	.33
Perceived social support (MOS-SSS^i^)										
0-6 mo, Δ	0.64 (−2.67 to 3.95)	.70	0.88 (−2.39 to 4.16)	.60	−0.38 (−3.70 to 2.93)	.82	0.75 (−2.65 to 4.16)	.66	1.83 (−1.41 to 5.06)	.27
0-12 mo, Δ	−0.03 (−3.35 to 3.33)	.99	2.89 (−0.40 to 6.22)	.09	1.22 (−2.15 to 4.55)	.48	4.76 (1.28-8.15)	.007	4.63 (1.32-7.85)	.006
**Hypothesized proximal outcome**										
Self-care risk (RAM-SC^j^)										
0-6 mo, Δ	0.77 (0.06-1.47)	.03	−1.12 (−1.82 to −0.43)	.002	0.50 (−0.20 to 1.20)	.16	−0.06 (−0.79 to 0.66)	.86	−0.49 (−1.18 to 0.20)	.16
0-12 mo, Δ	0.57 (−0.14 to 1.30)	.12	−0.60 (−1.31 to 0.12)	.10	0.75 (0.03-1.47)	.04	−0.25 (−1.00 to 0.48)	.50	0.00 (−0.71 to 0.70)	.99
Dementia care strategies (DMSS^k^)										
Criticism domain										
0-6 mo, Δ	0.04 (−1.78 to 1.85)	.97	0.91 (−0.89 to 2.71)	.32	−1.65 (−3.47 to 0.17)	.08	2.01 (0.14 to 3.88)	.04	0.65 (−1.13 to 2.43)	.47
0-12 mo, Δ	0.54 (−1.40 to 2.48)	.58	0.92 (−1.01 to 2.84)	.35	−0.96 (−2.91 to 0.98)	.33	−0.29 (−2.28 to 1.72)	.78	0.29 (−1.61 to 2.19)	.77
Encouragement domain										
0-6 mo, Δ	−0.09 (−1.74 to 1.55)	.91	0.29 (−1.34 to 1.92)	.72	−0.82 (−2.47 to 0.83)	.33	−0.95 (−2.65 to 0.74)	.27	0.64 (−0.97 to 2.25)	.44
0-12 mo, Δ	0.26 (−1.50 to 2.01)	.77	2.49 (0.74-4.22)	.005	0.34 (−1.42 to 2.10)	.71	1.75 (−0.04 to 3.57)	.06	−1.17 (−2.88 to 0.55)	.18
Active management domain										
0-6 mo, Δ	0.37 (−1.40 to 2.13)	.68	0.75 (−1.00 to 2.50)	.40	−0.80 (−2.57 to 0.97)	.37	1.21 (−0.60 to 3.03)	.19	1.34 (−0.38 to 3.07)	.13
0-12 mo, Δ	−0.21 (−2.10 to 1.66)	.83	5.99 (4.12-7.84)	<.001	−0.13 (−2.01 to 1.76)	.89	3.70 (1.80-5.66)	<.001	−1.07 (−2.88 to 0.78)	.25
Engagement in pleasurable activities, No. of meaningful events^l^										
0-6 mo, Δ	0.16 (−0.15 to 0.48)	.31	0.20 (−0.11 to 0.52)	.21	−0.23 (−0.55 to 0.09)	.16	0.03 (−0.29 to 0.36)	.85	−0.01 (−0.32 to 0.30)	.95
0-12 mo, Δ	0.14 (−0.18 to 0.46)	.39	0.29 (−0.03 to 0.61)	.07	−0.31 (−0.63 to 0.01)	.06	0.03 (−0.29 to 0.37)	.84	0.07 (−0.24 to 0.39)	.65
Trait mindfulness (FFMQ^m^)										
0-6 mo, Δ	−0.50 (−2.46 to 1.46)	.61	0.15 (−1.79 to 2.09)	.88	−1.55 (−3.52 to 0.41)	.12	1.13 (−0.89 to 3.14)	.27	0.87 (−1.04 to 2.79)	.37
0-12 mo, Δ	−0.49 (−2.52 to 1.46)	.63	1.98 (−0.04 to 3.90)	.05	−0.11 (−2.07 to 1.92)	.91	4.23 (2.27-6.36)	<.001	−1.01 (−2.88 to 1.00)	.30
Satisfaction with support group^n^										
6-12 mo, Δ	−0.11 (−1.09 to 0.85)	.82	−0.39 (−1.36 to 0.55)	.42	−0.17 (−1.13 to 0.81)	.73	1.97 (0.99-2.98)	<.001	−0.39 (−1.32 to 0.57)	.42

Abbreviations: DMSS, Dementia Management Strategies Scale; FFMQ, Five Facet Mindfulness Questionnaire; HADS-A, Hospital Anxiety and Depression Scale–Anxiety Subscale; MOS-SSS, Medical Outcomes Study Social Support Survey; PHQ-9, 9-item Patient Health Questionnaire; PSS, Perceived Stress Scale; PWB, Ryff Psychological Well-Being Scale; RAM-SC, Risk Appraisal Measure–Self-Care; SF-12, 12-item Short-Form Health Survey; ZBI, Zarit Burden Interview Scale.

^a^ All participants (N = 250) were analyzed according to allocation. Values are presented as estimated marginal mean changes unless indicated otherwise.

^b^ All *P* values reported in the analysis were adjusted for age, sex, educational attainment, number of components the participants had, number of years in caregiving, and whether the caregivers were the sole caregivers. To account for multiplicity, *P* < .01 (2-tailed) was considered statistically significant.

^c^ Higher scores (range, 0-100) indicate better physical health condition.

^d^ Higher scores (range, 0-48) indicate greater caregiver burden.

^e^ Higher scores (range, 0-40) indicate higher levels of stress.

^f^ Higher scores (range, 16-96) indicate better psychological well-being.

^g^ Higher scores (range, 0-21) indicate higher levels of anxiety.

^h^ Higher scores (range, 0-27) indicate greater severity of depressive symptoms.

^i^ Higher scores (range, 19-95) indicate higher levels of perceived social support.

^j^ Higher scores (range, 0-11) indicate lower perceived risk.

^k^ Categorized into 3 subscales of caregiving strategies (criticism, encouragement, and active management); higher scores indicate more frequent corresponding caregiving behaviors.

^l^ Number of meaningful events (range, 0-5); higher numbers indicate more meaningful events in the past week.

^m^ Measures mindfulness with regard to thoughts, experiences, and actions in daily life, (range, 20-100); higher scores indicate higher levels of mindfulness.

^n^ Measures satisfaction with the social support group (range, 5-35); higher scores indicate greater levels of satisfaction. The change in scores from 12 months to 6 months was compiled to assess the degree of change in satisfaction over time.

#### Hypothesized Proximal Outcomes

Following ITT analyses, Table 2 presents the main effects of the intervention components on the corresponding proximal outcomes over a 12-month period. For the hypothesized proximal outcomes, participants who received the BPM component reported a significant improvement in dementia management strategies as assessed with the DMSS–Encouragement (β = 2.49 [95% CI, 0.74-4.22]; *P* = .005) and DMSS–Active Management (β = 5.99 [95% CI, 4.12-7.84]; *P* < .001) domains at 12 months. The MBI component significantly improved trait mindfulness (FFMQ: β = 4.23 [95% CI, 2.27-6.36]; *P* < .001) at 12 months. Beyond the hypothesized proximal outcomes, BPM significantly increased the self-care risk of caregivers (RAM-SC: β = −1.12 [95% CI, −1.82 to −0.43]; *P* = .002) at 6 months; the MBI component yielded significant improvement in dementia management strategies as assessed with DMSS–Active Management domain (β = 3.70 [95% CI, 1.80-5.66]; *P* < .001) at 12 months and satisfaction with social support group (β = 1.97 [95% CI, 0.99-2.98]; *P* < .001).

### Exploratory Analyses of 2-Way Interaction Effects of Intervention Components

**Figure 2.**
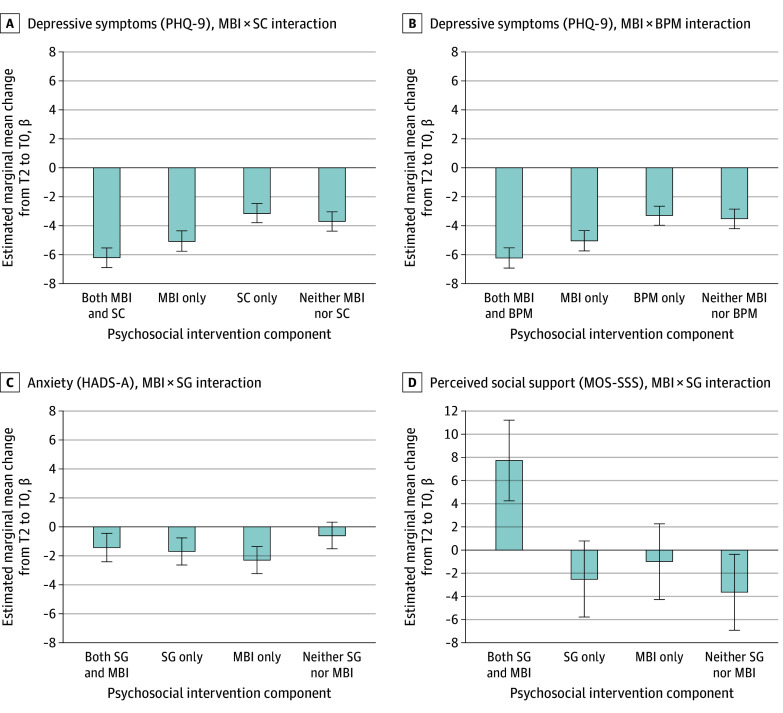
Contrast Analyses of Statistically Significant 2-Way Interactions That Aligned With the Corresponding Hypothesis Error bars represent 95% CIs for the estimated marginal means for each tested condition. BPM indicates behavioral problem management; HADS-A, Hospital Anxiety and Depression Scale–Anxiety Subscale; MBI, mindfulness-based intervention; MOS-SSS, Medical Outcomes Study Social Support Survey; PHQ-9, Patient Health Questionnaire-9; SC, self-care skills; SG, social support group; T0, baseline; T2, 12-month follow-up.

The exploratory analyses examined the main and 2-way interaction effects of the intervention components at 12 months. Nine statistically significant interactions were observed (Table 3). Figure 2 presents contrast analyses for the 4 significant interactions that aligned with the corresponding hypotheses. For the SC × MBI interaction (PHQ-9: β = −1.70 [95% CI, −3.05 to −0.35]; *P* = .01) (Figure 2A), participants who received MBI and SC showed greater reductions in depressive symptoms compared with those receiving MBI without SC, SC without MBI, and neither SC nor MBI. Similarly, the BPM × MBI interaction (PHQ-9: β = −1.40 [95% CI, −2.76 to −0.05]; *P* = .04) (Figure 2B) indicated that the combination of MBI and BPM resulted in the greatest reduction in depressive symptoms, followed by MBI without BPM. In the MBI × SG interaction (HADS-A: β = 1.98 [95% CI, 0.08-3.89]; *P* = .04) (Figure 2C), those receiving MBI without SG and SG without MBI demonstrated reductions in anxiety, yet the combination of SG and MBI did not result in further improvement. Finally, the MBI × SG interaction (MOS-SSS: β = 7.58 [95% CI, 0.90-14.26]; *P* = .03) (Figure 2D) revealed that participants receiving SG and MBI demonstrated improvement in perceived social support, whereas those receiving SG without MBI, MBI without SG, and neither SG nor MBI reported worsened social support.

**Table 3.** Adjusted Regression Analyses for Change in Primary Outcomes of 5 Components and the 2-Way Interactions

Component	Psychosocial component, primary outcome (assessment tool)^a^													
	Self-care skills, physical health (SF-12)		Behavioral problem management				Behavioral activation, psychological well-being (PWB)		Mindfulness-based intervention				Social support group, perceived social support (MOS-SSS)	
			Caregiving burden (ZBI)		General stress (PSS)				Anxiety (HADS-A)		Depressive symptoms (PHQ-9)			
	β (95% CI)	*P* value^b^	β (95% CI)	*P* value	β (95% CI)	*P* value	β (95% CI)	*P* value	β (95% CI)	*P* value	β (95% CI)	*P* value	β (95% CI)	*P* value
SC	2.21 (−0.99 to 5.40)	.18	2.74 (−1.30 to 6.78)	.18	2.66 (−0.18 to 5.50)	.07	3.61 (−2.00 to 9.22)	.21	−0.93 (−3.03 to 1.17)	.38	1.39 (−0.10 to 2.89)	.07	1.78 (−5.61 to 9.16)	.64
BPM	1.72 (−1.47 to 4.91)	.29	0.28 (−3.76 to 4.32)	.89	0.90 (−1.94 to 3.74)	.53	7.16 (1.55-12.77)	.01	−0.12 (−2.22 to 1.98)	.91	0.47 (−1.03 to 1.96)	.54	0.21 (−7.18 to 7.59)	.96
BA	−1.43 (−4.62 to 1.77)	.38	0.07 (−3.98 to 4.11)	.97	0.92 (−1.93 to 3.76)	.53	1.12 (−4.49 to 6.73)	.70	−0.19 (−2.29 to 1.91)	.86	0.38 (−1.12 to 1.87)	.62	5.12 (−2.26 to 12.51)	.17
MBI	2.69 (−0.51 to 5.88)	.10	3.48 (−0.56 to 7.52)	.09	0.35 (−2.49 to 3.20)	.81	5.76 (0.15-11.37)	.04	−1.97 (−4.07 to 0.13)	.07	0.50 (−0.99 to 2.00)	.51	4.41 (−2.97 to 11.79)	.24
SG	−1.22 (−4.41 to 1.97)	.45	−0.32 (−4.37 to 3.72)	.88	−0.89 (−3.73 to 1.95)	.54	−1.24 (−6.85 to 4.37)	.66	−2.60 (−4.70 to −0.50)	.02	0.74 (−0.75 to 2.23)	.33	−0.37 (−7.75 to 7.01)	.92
SC × BPM	0.10 (−2.79 to 2.98)	.95	−0.88 (−4.54 to 2.77)	.64	−1.44 (−4.01 to 1.13)	.27	−1.81 (−6.89 to 3.27)	.48	0.53 (−1.37 to 2.43)	.58	−1.31 (−2.66 to 0.04)	.06	4.32 (−2.36 to 11.01)	.20
SC × BA	−0.64 (−3.53 to 2.25)	.66	−3.24 (−6.90 to 0.42)	.08	−3.39 (−5.97 to −0.82)	.01	−2.65 (−7.73 to 2.42)	.30	−0.61 (−2.51 to 1.29)	.53	−1.02 (−2.37 to 0.33)	.14	−3.74 (−10.42 to 2.94)	.27
SC × MBI	−1.93 (−4.82 to 0.96)	.19	−3.21 (−6.87 to 0.45)	.09	0.92 (−1.65 to 3.49)	.48	0.77 (−4.30 to 5.85)	.76	0.62 (−1.28 to 2.52)	.52	−1.70 (−3.05 to −0.35)	.01	2.39 (−4.29 to 9.07)	.48
SC × SG	−1.44 (−4.33 to 1.45)	.33	3.93 (0.27-7.59)	.04	0.42 (−2.16 to 2.99)	.75	−2.99 (−8.06 to 2.09)	.25	1.96 (0.06-3.86)	.04	0.66 (−0.69 to 2.01)	.34	−4.65 (−11.33 to 2.03)	.17
BPM × BA	−0.69 (−3.58 to 2.02)	.64	1.00 (−2.66 to 4.66)	.59	−0.37 (−2.94 to 2.20)	.78	1.86 (−3.22 to 6.94)	.47	0.20 (−1.70 to 2.11)	.83	0.99 (−0.36 to 2.35)	.15	−1.24 (−7.92 to 5.44)	.72
BPM × MBI	−3.81 (−6.70 to −0.92)	.01	−2.15 (−5.81 to 1.51)	.25	−1.16 (−3.73 to 1.41)	.38	−7.35 (−12.42 to −2.27)	.005	−0.78 (−2.68 to 1.12)	.42	−1.40 (−2.76 to −0.05)	.04	−1.17 (−7.85 to 5.52)	.73
BPM × SG	1.90 (−0.99 to 4.79)	.20	0.35 (−3.31 to 4.01)	.85	0.94 (−1.63 to 3.51)	.47	0.61 (−4.47 to 5.69)	.81	1.48 (−0.42 to 3.38)	.13	−0.17 (−1.53 to 1.18)	.80	4.48 (−2.20 to 11.17)	.19
BA × MBI	0.96 (−1.93 to 3.85)	.51	0.91 (−2.75 to 4.57)	.62	0.76 (−1.81 to 3.33)	.56	−1.45 (−6.53 to 3.62)	.57	0.67 (−1.23 to 2.57)	.49	−1.16 (−2.51 to 0.19)	.09	−4.69 (−11.38 to 1.99)	.17
BA × SG	0.26 (−2.62 to 3.15)	.86	−1.60 (−5.25 to 2.06)	.39	−1.29 (−3.86 to 1.29)	.33	2.47 (−2.61 to 7.54)	.34	−0.46 (−2.36 to 1.44)	.64	−0.13 (−1.48 to 1.23)	.86	3.19 (−3.49 to 9.87)	.35
MBI × SG	1.63 (−1.26 to 4.52)	.27	0.31 (−3.35 to 3.97)	.87	0.55 (−2.03 to 3.12)	.68	3.03 (−2.04 to 8.11)	.24	1.98 (0.08-3.89)	.04	−1.17 (−2.52 to 0.18)	.09	7.58 (0.90-14.26)	.03

Abbreviations: BA, behavioral activation; BPM, behavioral problem management; HADS-A, Hospital Anxiety and Depression Scale–Anxiety Subscale; MBI, mindfulness-based intervention; MOS-SSS, Medical Outcomes Study Social Support Survey; PHQ-9, 9-item Patient Health Questionnaire; PSS, Perceived Stress Scale; PWB, Ryff Psychological Well-Being Scale; SC, self-care skills; SF-12, 12-item Short-Form Health Survey physical health component; SG, social support group; ZBI, Zarit Burden Interview Scale.

^a^ All participants (N = 250) analyzed according to allocation. Values are presented as estimated marginal mean changes from 12 months to baseline unless indicated otherwise.

^b^ All *P* values reported in the analysis were adjusted for age, sex, educational attainment, number of components the participants had, number of years in caregiving, and whether the caregivers were the sole caregivers.

## Discussion

Empirical evidence to guide the development of efficient and scalable dementia caregiving programs is urgently needed. This fractional factorial optimization trial investigated which of the 5 components of a remotely delivered, technology-supported caregiving program contributed mainly to supporting informal dementia caregivers who experienced moderate depressive symptoms or caregiving burden. To our knowledge, this study is the first application of the MOST framework to the caregiving field and provides valuable insights to inform future resource allocation and health policy decisions. Execution of the 5-factor design was feasible and identified several promising effects from the individual intervention components. Empirical insights from the component interactions were obtained. The findings suggest that integrating mindfulness with support group or behavioral management components may be an effective multicomponent approach, although ongoing support is needed to mitigate potential short-term risks.

The statistically significant main effects of the individual intervention components observed in this study align with prior research on the benefits of mindfulness-based approaches, support groups, and behavioral or psychoeducational management on mental health and caregiving skills.^32,33,34^ Specifically, in our study, the MBI component significantly reduced depressive symptoms and improved trait mindfulness, perceived social support, active dementia management strategies, and satisfaction with the support group at the 12-month follow-up. The SG component significantly improved perceived social support at 12 months. The BPM component increased anxiety and self-care risk at the 6-month time point but significantly improved dementia management strategies and psychological well-being at 12 months. The initial increase in caregiver anxiety and reduced self-care from the BPM component likely reflects the short-term challenges of learning new behavioral management skills, resulting in less time and capacities for self-care among caregivers.^35^ This delayed benefit suggests the importance of ongoing personalized support and monitoring to facilitate the mastery of behavior modification skills into tangible improvements for caregiver well-being.

The interaction effects observed in this study offer further insights into potential mechanisms. The synergistic effects of MBI with SC and BPM on depressive symptoms suggest that mindfulness techniques may amplify the benefits of building specific self-care abilities or managing behavioral issues. The SC component was designed to help caregivers to be more aware of their own well-being and engage in healthy lifestyle behavior, whereas the BPM component focused on identifying, evaluating, and changing problematic behavior exhibited by care recipients. Mindfulness can enhance self-regulation, reduce cognitive distortions, promote self-compassion, and facilitate present-moment focus,^34,36^ all of which may allow caregivers to more effectively apply the practical skills and strategies learned in the SC and BPM components. This finding highlights the potential for mindfulness to boost the effectiveness of caregiver interventions by cultivating the psychological resources that facilitate the integration and application of targeted skills.^37,38^

Another promising synergistic effect was noted between the MBI and SG components on perceived social support. MBI can enhance social connection and emotional regulation,^36^ whereas the SG component may have provided a safe, shared space for caregivers to express vulnerabilities, engage with mindfulness, and foster a sense of belonging.^39,40^ This mutual reinforcement between mindfulness-enhanced social capacities and the tangible support offered by the group setting leads to a greater sense of social support for caregivers compared with each component alone.

However, such benefits may not extend to caregivers’ anxiety outcomes. A statistically significant antagonistic interaction was found between the SG and MBI components in terms of anxiety reduction. One potential explanation is that the group dynamic and interpersonal sharing during SG could trigger worry, rumination, or social anxiety, which may counteract the calming, detached mindset developed through mindfulness.^41^ As such, when integrating MBI and SG, facilitators should set clear agreements for mindful engagement.^42^ Efforts should be made to cultivate a respectful beginner’s mindset, avoid blaming or shaming, acknowledge intent vs impact, honor differences in perspectives, maintain confidentiality, and approach interactions with compassion, to mitigate any adverse effects on anxiety when combining these therapeutic components.

It is surprising that the BA component did not result in any statistically significant caregiver outcomes. Factors such as cognitive demands of caregiving, lack of personalization of the intervention, competing priorities, and the practical problem-solving orientation of Chinese caregivers may have influenced their experiences and engagement in pleasurable activities.^43^ Future BA interventions could consider incorporating a peer-led approach,^44^ in which shared feelings and experiences could complement skill-building and help caregivers apply techniques to their unique contexts.

### Strengths and Limitations

This study has several strengths. This research highlights the value of the MOST approach. In particular, the fractional factorial design allowed for the screening of 5 unique intervention components in a single experiment. The acceptability, participation, and satisfaction of the 5 remotely delivered, technology-support psychosocial components are well established for a range of caregiving outcomes. These findings demonstrate success in adopting a pragmatic approach to the development of intervention components in which all components, durations, and delivery systems were designed for application in community settings while keeping the duration as short as possible.^21^ In this article, we have made a deliberate and somewhat unconventional decision to present all primary outcome findings for the 5 tested components in a single comprehensive report. This approach avoids selectively reporting only positive outcomes or disseminating partial findings across multiple publications. By providing a complete view of all outcomes, our study paves the way for future research on outcome and intervention component selection. This holistic perspective enhances understanding of what constitutes the so-called optimal intervention for different groups, subgroups, or individuals, ultimately increasing the effectiveness and impact of interventions tailored to diverse needs.

However, this study has several limitations that warrant acknowledgment. One notable limitation is related to our analytical approach, which used separate multiple linear regression models with change scores as dependent variables. Although this method effectively addressed our immediate research needs, it may not fully capture the complexities of the data. Linear mixed models (LMMs) could provide advantages such as improved management of missing data, better accounting for individual variability in temporal changes, and a more comprehensive perspective on longitudinal relationships.^45^ Nonetheless, given our study’s retention rate of greater than 94.0%, any potential bias from missing data is likely negligible. Our preference for multiple regression was based on its simplicity and interpretability, particularly in the context of high data integrity. This method also proved effective in handling combinations of 2 intervention components. However, LMMs can face challenges when modeling complex interactions among intervention components; specifically, we encountered convergence issues and model instability when attempting to incorporate multiple interaction terms within the LMM framework. To further validate our main findings, we conducted parallel analyses using LMMs for the main effects of both primary and proximal outcomes (eTables 3 and 4 in Supplement 2), which yielded results comparable to our regression analyses, reinforcing the robustness and validity of our chosen methodologic approach. Additionally, variability in the dosage of intervention components received by participants represents another limitation that could affect the effectiveness of the interventions. Although the analyses were adjusted for the number of components the participants received, future studies could consider testing intervention components with similar dosage and delivery settings to minimize confounding factors. Furthermore, the MBI component was delivered by a mindfulness instructor, and the SG component was facilitated by social workers—professionals with existing expertise in the field. In contrast, other intervention components were led by trained research assistants, who, while capable, may not have had the same level of expertise. This disparity in leadership could have influenced the positive effect of mindfulness and support group interventions. Finally, we recommend conducting a well-designed RCT to definitively test the efficacy of integrated intervention components, explore underlying mechanisms behind observed interactive effects, assess generalizability, and investigate methods to mitigate potential adverse effects on anxiety reduction when combining group support and mindfulness-based components. These approaches would enhance the ability to detect meaningful effects, provide more robust insights, and address the limitations identified in the current study.

## Conclusions

In this randomized clinical trial of informal caregivers of people with dementia, synergistic interaction effects were noted for mindfulness, enhancing the benefits of self-care and BPM on depression. Mindfulness and group support synergistically improved social support. Our findings demonstrate the value of using the MOST framework with a fractional factorial design to guide the development of efficient dementia caregiver programs. Inclusion of the MBI and SG components emerged as a parsimonious yet effective multicomponent intervention. The approach yielded benefits such as reduced depression, improved social support and mindfulness traits, and active dementia management and satisfaction with social support group, which appeared to work well together. If resources allow, these findings suggest that incorporating BPM could further improve caregivers’ dementia management skills and psychological well-being in the long run. However, ongoing support was crucial throughout the skill learning process to avoid inducing short-term anxiety and self-care risks. Finally, the combination of MBI with SC or BPM components appeared promising for managing caregiver depression. The research found interaction effects and highlighted the need to evaluate both main and interaction effects to systematically identify essential components and enable personalization. Although the identified components showed promise, an RCT is required to validate their efficacy as an integrated treatment package. These findings have substantial implications for the strategic, evidence-based design and implementation of effective dementia care programs aimed at empowering caregivers, improving their coping, and fostering a supportive environment.

## Notes

Supplement 1.
Trial Protocol

Supplement 2. **eTable 1.** Experimental Conditions of the Fractional Factorial Randomized Clinical Trial **eFigure 1.** Supposed Impact of the Different Intervention Components on the Stress–Health Process of the Informal Caregiver **eFigure 2.** Mechanism of the Multicomponent Intervention **eTable 2.** Satisfaction Level of Family Dementia Caregivers by Each Component (N = 250) **eTable 3. **Analysis of Main Effects of Primary Outcomes Corresponding to Each Tested Component Using the Mixed Models at Baseline, 6 Months of Follow-Up, and 12 Months of Follow-Up **eTable 4.** Analysis of Main Effects of Proximal Outcomes Using the Mixed Models at Baseline, 6 Months of Follow-Up, and 12 Months of Follow-Up

Supplement 3.
Data Sharing Statement
