# Supplementary material for: Multicomponent Intervention for Distressed Informal Caregivers of People With Dementia: A Randomized Clinical Trial
Source: JAMA Netw Open. 2025 Mar 17;8(3):e250069. doi: 10.1001/jamanetworkopen.2025.0069 (PMC11915064; doi:10.1001/jamanetworkopen.2025.0069)
Supplement: Supplement 1. — Trial Protocol [file jamanetwopen-e250069-s001.pdf]

Supplement 1: Study Protocol

Full title

Multiphase optimization of a multicomponent intervention for informal dementia caregivers: A study protocol

ABSTRACT

Background

Family caregiver interventions are essential to support dementia caregiving. However, such interventions are typically complex and consist of multiple components. Existing evidence rarely delineate the effectiveness and interactions between individual components. To optimise intervention, we adopt the multiphase optimisation strategy (MOST) to test the implementation fidelity and determine the effect of each component and the interactions between each component and the corresponding outcome.

Methods

A prospective, assessor-blinded, randomised clinical trial with fractional factorial design using the MOST principle. 250 family dementia caregivers will be randomised to one of 16 experimental conditions in a fractional factorial design involving six intervention components: 1) dementia and caregiving education; 2) self-care skills; 3) behavioural symptom management; 4) behavioural activation; 5) modified mindfulness-based cognitive therapy; and 6) support group. The first one is the core component, and the five remaining will be examined. Physical health, caregiver burden, stress, psychological well-being, anxiety and depressive symptoms, and social support will be assessed over the 12-month study period. Following the intention-to-treat principle, linear mixed models and regression analyses will be used to examine the specific effect of the five components and their two-way interactions to propose the most effective combination.

Discussion

This is the first study adopting the multiphase optimisation strategy to identify the most active and engaging components of a psychological intervention for caregivers of patients with dementia. In view that dementia caregiver interventions are increasingly diversified and complex, such knowledge is important to maximise the intervention efficacy and allow the intervention to be implemented within an efficient timeframe and dosage. The optimisation of caregiver support interventions is critical to enhance the health outcomes of caregivers and care recipients, thereby, delaying possible institutionalization and reducing the costs of long-term dementia care.

Trial Registration

This study was retrospectively registered in the WHO Primary Registry – Chinese Clinical Trials Registry (ChiCTR2300071235). (Protocol date 30/10/2020; version identifier 2020-2021-0045). Registered 9 May, 2023. https://www.chictr.org.cn/showprojEN.html?proj=194600

Reporting Method

SPIRIT guideline was followed.

Patient or Public Contribution

No patient or public involvement.

Keywords

Dementia caregiver, multicomponent, caregiving burden, depressive symptoms, positive aspects of, randomized controlled trial, REACH, Multiphase Optimization

INTRODUCTION

Dementia is an impending healthcare crisis that affects more than 55 million individuals worldwide. With the aging population, this number is expected to reach approximately 78 million by 2030 and 139 million by 2050 (1). The global financial burden of dementia is estimated to be USD 1.3 trillion and may reach USD 2.8 trillion by 2030. Dementia is a progressively degenerating disease that causes multiple cognitive deterioration, which results in disability, dependence, institutionalisation and mortality. Long-term care of patients with dementia is mostly provided by family caregivers, who provide unpaid or informal attention and play an essential role for the persons they are taking care of and for the overall healthcare system. Family dementia caregivers are more stressed, suffer more serious anxiety and depressive symptoms and are at higher risk of cardiovascular diseases than non-dementia caregivers (2). These over-burdened caregivers are more likely to give up their caregiver role by institutionalisation of their care recipient (3). All of these factors are projected to significantly increase the costs of healthcare and long-term care in the future. Therefore, effective interventions should be designed to reduce the negative effects of caregiving on family dementia caregivers.

A recent systematic review and meta-analysis compared the effects of 131 randomised controlled trials (RCTs) for dementia caregivers with community-dwelling care recipients; the result showed that multicomponent interventions had the most significant effect among nonpharmacological interventions on reducing the burdens on family dementia caregivers, decreasing their stress levels and enhancing their subjective well-being (4). An earlier meta-analysis reported that multicomponent interventions are effective in delaying or preventing the institutionalisation of people with dementia (5). Despite the favourable outcomes of multicomponent interventions, the effectiveness of individual components is concealed with the black box approach in traditional RCTs. Caregiving support interventions are often complex and consist of multiple components in variable combinations. For example, an intervention may include components, such as education on dementia and caregiving education, self-care skills, behavioural symptom management, stress management and support group. Traditional RCTs have only examined the effectiveness of various combinations of interventions as a package. The effectiveness of individual components of a multicomponent intervention has been rarely tested. As such, studies of broadly conceived family caregiver programs consisting of different components have produced highly varied results in terms of effect size, which ranges from small to medium (4). Components and behavioural skills that can effectively alleviate caregiving burden and enhance psychosocial outcomes among dementia family caregivers and to whom they are suitable for remain to be identified.

The multiphase optimisation strategy (MOST) developed by Collins and her colleagues (6) is an innovative approach for examining which components of a multicomponent intervention are effective in achieving a particular outcome, singly and in combination with one another. The value of MOST has been established in recent research in several areas, including cessation of smoking, promotion of physical activity, reducing obesity, reducing alcohol use and unsafe sex and conducting education interventions (7). However, to our knowledge, MOST has not been applied in gerontology, making our proposed study the first of its kind. MOST consists of three stages: 1) preparation to conduct an optimisation trial, 2) optimisation to reveal what constitutes an optimised intervention and 3) evaluation of the optimised intervention relative to an established intervention in an RCT. In this study, we will focus only on the preparation and optimisation phases due to resource and time constraints.

In the preparation phase, we will use six core psychoeducational components, namely, 1) education on dementia and caregiving, 2) self-care skills, 3) behavioural symptom management, 4) behavioural activation, 5) modified mindfulness-based cognitive therapy and 6) social support. Components 1 to 4 and 6 were adapted from the ‘Resources for Enhancing Alzheimer’s Caregiver Health’ (REACH) intervention (8-10), which is one of the most studied multicomponent interventions for informal dementia caregivers. Two multicomponent intervention studies of REACH that considered cultural perspective in caregiving (11) were conducted in Hong Kong and reported positive preliminary findings. In the 2010 study (9), Au adapted and evaluated the multicomponent intervention called Coping with Caregiving, which is one version of REACH; they found that the intervention enhanced caregiving self-efficacy and

effective coping strategies. In the 2015 study (10), Cheung conducted an implementation study of the translated version of REACH II by using a quasi-experimental single-group pre−post treatment design. They found that it had significant effects on the perception of positive aspects of caregiving, reduction in depressive symptoms, subjective burden, bother and caregiving risks among caregivers and abatement in behavioural problems among care recipients. Promises in reach and adoption were demonstrated by the participation of 85 interventionists from 11 non-governmental organisations across 18 districts and 243 caregivers of various demographic characteristics. In a 2020 meta-analysis (4), multicomponent interventions for dementia caregivers had limited positive effect on depressive symptoms. By contrast, mindfulness-based interventions significantly reduced anxiety and depressive symptoms (4, 12-15). Hence, this proposed study will incorporate the modified mindfulness-based cognitive therapy, which has been tested effectively among dementia caregivers, as a stress management component (16, 17).

Following the MOST framework, we will select the treatment components carefully to ensure that they are conceptually and operationally distinct from one another, so each component can be evaluated independently. However, we do not assume that the treatment components do not interact. Interaction effects may exist between individual components and influence primary outcomes. For example, during support group sessions, caregivers may exchange skills and information on managing the behavioural symptoms of care recipients by sharing and discussion; as such, the caregivers may build strong bonds and a sense of social support. As a result, behavioural symptom management component may moderate and strengthen the relationship between support group component and perceived social support. In this regard, social support had a significant effect only when incorporated in multicomponent interventions instead of other domain-specific interventions (18). Therefore, in the optimisation phase, we will use a factorial approach with effect coding to concurrently test the main effects of individual components and their interaction effects. All experimental conditions will involve education on dementia and caregiving as the core component, since it has been identified as the most ubiquitous component for caregiver support programmes as well as its ease of implementation. This factional factorial design will reduce the total number of conditions from 32 to 16, which retains the benefits of a factorial design whilst allowing a more logistically manageable and feasible study.

Another major limitation of studies on multicomponent interventions is that researchers often omit to report whether components have been implemented with fidelity; thus, the implementation fidelity of individual components remains unknown (19). Assessment of implementation fidelity is an important first step in determining the effectiveness of a treatment component. Moreover, to our knowledge, no previous study has examined the underlying mechanism of individual components of multicomponent interventions. The factorial design of single intervention components will enable us to determine not only which components are the most strongly associated with changes in primary outcomes but also the fidelity of the implementation of each component, the components that lead to improvements in corresponding proximal outcomes (i.e. the goal of the component) and whether the proximal outcomes mediate the effects of their corresponding components on primary outcomes (i.e. understand the underlying mechanism of each component).

Multicomponent interventions can be tailored to meet the specific needs of individual caregivers given the variability inherent in caregiving situations (8). Specific treatment modules or strategies and techniques may be applied according to the risk profiles of caregivers to provide personalised support. However, whether such matching between the risk profiles of caregivers and individual components works better than a one-size-fits-all approach remains unknown because the relative efficacy of a specific component on a specific outcome has yet to be examined systematically. Hence, this study will also determine the moderating effects of the baseline scores of primary outcome measures in the relationship between intervention components and primary outcomes.

Although theoretical and empirical evidence suggests the positive effects of multicomponent interventions, a limited number of dementia caregiving support interventions have looked into the "black box" to understand which intervention components work or do not work, and how they work. To optimize the efficacy and scalability of

multicomponent interventions, investigations should cover implementation fidelity, the main effect of individual components and their interactions and the underlying mediating/moderating mechanism. This study aims to address these research gaps. The findings will provide a wider repertoire of evidence-based personalised multicomponent interventions to support dementia caregivers.

Objectives

This study aims to:

1. determine the implementation fidelity of each intervention component,
2. assess the effect of each component on corresponding proximal outcomes,
3. examine the effect of each component on primary outcomes (e.g. depression, burden),
4. determine if the proximal outcomes of each component mediate the components' effects on primary outcomes,
5. determine the interaction effect between components on their proximal and primary outcomes and
6. determine the moderating effect of the baseline scores of primary outcomes in the relationship between each intervention component and the corresponding outcome.

The hypotheses to be tested are as follows:

1. The implementation fidelity of all six components is equally high.
2. Components 2 (self-care skills), 3 (behavioural symptom management), 4 (behavioural activation), 5 (modified mindfulness-based cognitive therapy) and 6 (support group) will have significant effects on proximal outcomes, namely, self-care, dementia caregiving strategy, engagement in pleasurable activity, mindfulness and satisfaction with support group, respectively.
3. Components 2–6 will have a stronger effect on their corresponding primary outcomes, namely, physical health status, stress/burden, psychological well-being, anxiety/depressive symptoms and social support, respectively, than the other components.
4. Self-care, dementia caregiving strategy, engagement in pleasurable activity, mindfulness and satisfaction with support group mediate the relationship between component 2 and physical health status, between component 3 and stress/caregiving burden, between component 4 and psychological well-being, between component 5 and anxiety/depressive symptoms and between component 6 and social support, respectively.
5. Component 3 moderates the relationship between component 6 and social support.
6. The baseline scores of physical health status, stress/burden, psychological well-being, anxiety/depressive symptoms and social support moderate the effects of components 2–6 on changes in scores between the baseline and follow-up assessments of the corresponding outcomes.

Trial design

This study adopts a prospective, assessor-blinded, randomised controlled trial with fractional factorial design by using the MOST principles to evaluate the effects of the five individual components and their two-way interactions (including self-care skills, behavioural problem management, behavioural activation, mindfulness yoga and support group; Table 1). A complete factorial experiment of the five factors would have $2^5 = 32$ experimental conditions (ECs). To conserve resources and reduce logistical complexity, we select a 25-1 fractional factorial design and decrease the number of ECs from 32 to 16 (20). Our fractional factorial design is made up of a strategically selected subset of 32 ECs based on prioritising the estimation of intervention component main effects and two-way interactions (21). All the included ECs are listed in Table 1. The study contains a subset of ECs, so the participants will not be randomised into a usual care/control group but every EC can be served as a control condition under different circumstances to test the effect of each component. For example, the effect of component 2 on primary outcomes could be examined through the comparison of participants in EC1 to EC8 and those in EC9 to EC16. In addition, the study assumes the presence

of overlapping components within the 16 ECs. These overlaps will be recognised and carefully measured through fidelity monitoring. This study will conform with the Declaration of Helsinki.

Methods: Participants, interventions and outcomes

Study participants

Eligible family caregivers will be recruited if they satisfy the following inclusion criteria: (i) Hong Kong Chinese citizens aged 18 years or above; (ii) spouse, adult child or child-in-law of a care recipient; (iii) without cognitive impairment (i.e. HK-MoCA 5-Min with a cut-off score that is respective to the age and educational level); (iv) primary family caregiver for an individual with dementia (at least 20 hours per week) for at least one year; individuals are considered as caregivers if they have assisted with the activities of daily living (ADLs) and instrumental activities of daily living (IADLs) of people with dementia; and (v) caregivers with a certain degree of depression or feeling of burden (Patient Health Questionnaire-9 > 9 or Zarit Burden Scale >18) to ensure a homogeneous sample (22, 23).

Study setting, sampling and recruitment strategies

We will reach potential participants by using traditional and social-networking recruitment strategies. Traditional methods include newspaper and radio advertisements as well as referrals made through psychiatric and psychogeriatric clinics, non-governmental organisations providing elderly community services, Alzheimer's associations and university campuses. Social-networking methods include regular postings on sites, such as Facebook, and contextual targeting methods for identifying and directly targeting potential participants (based on their social media comments) with recruitment advertisements.

Allocation concealment, assignment of interventions and blinding

After baseline assessment, all participants will receive component 1 (dementia and caregiver education) as the core intervention. Participants will be randomly assigned to one of the 16 ECs (Table 1) by using the fractional factorial MOST design. Computer-generated random numbers will be used for allocation. Participants will be informed of their assigned group by an independent person not involved in the assessment. The allocation list will be computer-generated by an independent researcher and concealed from other researchers and participants until the time of assignment. Outcome assessors will be blinded to participant group allocation. Unblinding should only occur for participant safety concern when a series adverse event transpires and knowledge of the assignment of intervention could mitigate the health risk.

Figure 1. ***SPIRIT** figure - Schedule of enrolment, interventions, and assessments.*

	**STUDY PERIOD**														
	**Enrolment**	**Allocation**	**Post-allocation (months)**												**Close-out**
**TIMEPOINT**	-1	0						1						2	2
**Months**			1	2	3	4	5	6	7	8	9	10	11	12	
**ENROLMENT:**															
**Eligibility screen**	X														
**Informed consent**	X														
**Baseline assessment**	X														
**Allocation**		X													
**INTERVENTIONS:**															
All 16 experimental conditions (vary from 6-16 weeks)															
**ASSESSMENTS:**															
All variables								X						X	X

Components of the multicomponent intervention

In the MOST preparation phase, we develop a conceptual model (Figure 2) based on the theoretical framework model for the stress-health process of informal caregivers for PWDs (24). Figure 3 illustrates how each individual component affects its proximal outcome, which in turn affects the primary outcome.

<u>Component 1. Dementia and caregiving education.</u> This core component will provide general knowledge about dementia, information on skills for communicating with PWDs, common problems related to caregiving and seeking help. In line with the methods employed by Kwok (2013), three 30-minute sessions will be delivered via telephone and information will be provided in the form of presentation slides (also available in audio and video formats). Following safety walkthroughs (via Zoom) during the first two sessions, advice will be offered about ensuring home safety (e.g., through home modifications).

<u>Component 2. Self-care skills.</u> Participants will receive information related to the importance of self-care and its associated skills. This information will be provided in the form of presentation slides (also available in audio and video formats). Caregivers will be reminded of the importance of maintaining good health and continuing healthy behaviour for themselves and their care recipients. They will also be taught to use health passports to keep records of health conditions and doctor appointments of themselves and their care recipients.

Component 3. Behavioural symptom management. This component is adopted from previous studies conducted by our team members (26, 27), which will be delivered via telephone by well-trained research assistants. The antecedents–behaviour–consequence model (Teri et al., 1997) will be introduced to caregivers to manage the behavioural symptoms of care recipients. They will be taught to observe and record the antecedents and consequences of problem behaviour and to use weekly records to keep track of behavioural changes. They will also be taught how to develop plans to change precipitating events or their reactions to these problem behaviours. Home practices will be introduced, and caregivers will be encouraged to complete them regularly.

Component 4. Behavioural activation. In this component, we will encourage participants to schedule their engagement in pleasant activities into their daily routines by following the telephone-based protocol used in previous studies of Chinese caregivers conducted by our team members (26, 27). Specifically, caregivers will learn about the principles of BA and participate in sessions on activity monitoring, activity scheduling, reinforcing or modifying a pleasant event and activity rescheduling based on changes after modification.

Component 5. Modified mindfulness-based cognitive therapy. The programme will be led by a mindfulness practitioner and consists of seven 2-hour group sessions to be delivered online via video-conferencing software. Each session will have a theme and related activities. For instance, in the first session, the theme is waking up from automatic pilot and participants will be required to scan their body and perform raisin-eating meditation.

Component 6. Support group. This component consists of six one-hour group sessions delivered online, in which six major themes will be covered (28): introduction of dementia caregiving and development of a mutual support group; improvement of home care skills and interpersonal relationships; awareness of caregivers' mental health; reaching out to community resources; experience sharing on behavioural symptom management; and conclusion and review. These sessions will involve sharing information and engaging participants in group discussion as well as the provision of psychological support and participation in problem-solving exercises. An experienced social worker will act as a facilitator, and two peer leaders will be elected by group members.

Outcome measures

All outcome measures have been validated in local context and will be used in participants. Figure 3 lists all the primary and proximal outcomes.

Primary outcome measures

Physical health status will be measured by the validated 12-item Short-Form Health Survey (SF-12)(29). SF-12 consists of eight domains, and participant's health condition is rated on a 4-point Likert scale. Only the self-rated and physical component will be used to measure the primary outcome, and a high score indicates a good health condition. The Cronbach's alpha of the physical component in Chinese version is 0.68 (30).

Caregiver burden and general stress will be measured by the 12-item Zarit Burden Interview scale (ZBI) (31) and the 10-item Perceived Stress Scale (PSS) (32). ZBI uses a 5-point Likert scale, and a high score indicates a high caregiver burden. The Cronbach's alpha of the validated Chinese version is 0.84 (31). PSS consists of the positive element and negative element subscale and measures the subjective perception of stress in a 5-point Likert scale. A high score indicates a high stress level. The Cronbach's alpha is 0.75 (0.82 for negative subscales; 0.87 for positive subscale) (33).

Psychological well-being will be measured by the four subscales in Ryff's Psychological Well-Being Scale in 24-item version (34): self-acceptance (4 items), positive relations to others (4 items), purpose in life (4 items) and personal growth (4 items). The 16 items of subjective psychological well-being will be measured on a 6 point-Likert scale. The Cronbach's alpha of the four subscales ranges from 0.77 to 0.88 (34).

Anxiety and depressive symptoms will be measured by the 7-item Chinese version of the Anxiety Subscale of Hospital Anxiety and Depression Scale (HADS-A) (35) and the 9-item Chinese version of the Patient Health Questionnaire (PHQ-9), respectively (22). HADS-A uses a 4-point Likert scale to measure anxiety symptoms over the past week. The Cronbach's alpha is 0.77 for the anxiety subscale. PHQ-9 uses a 4-point Likert scale to assess depressive symptoms over the past week (36). A high score indicates severe depressive symptoms. The Cronbach's alpha is 0.91 (37).

Perceived adequacy of functional social support will be measured by the 20-item Medical Outcomes Study Social Support Survey (MOS-SSS) (38). MOS-SSS measures function aspects of perceived social support in four domains by using a 5-point Likert scale: tangible support, emotional-information support, positive social interactions and affectionate support. The Cronbach's alpha of the overall scale is 0.98 (39).

Proximal outcomes

Self-care will be measured by the 14-item self-care subscale in Risk Appraisal Measure (RAM-SC) (40, 41). The subscale illustrates the unique risk profile of caregivers in terms of self-care. A high score indicates a low risk of caregivers.

Caregiving strategies will be measured by the 34-item Dementia Management Strategies Scale (DMSS) (40, 41). The scale uses a 5-point Likert scale and consists of three domains, namely, criticism, encouragement and active management. The Cronbach's alpha of the three subscales ranges from 0.86 to 0.90 (40, 41).

Engagement in pleasurable activities will be measured by recording the frequency (times per week) and duration (minutes in each time) of each engagement in the past two weeks.

Mindfulness will be measured by the 20-item version of Five Facet Mindfulness Questionnaire (FFMQ) (42). Using a 5-point Likert scale, it measures five facets of mindfulness, namely, observing (4 items), describing (4 items), acting with awareness (4 items), nonjudging to inner experience (4 items) and nonreacting to inner experience (4 items). A high score reflects a high level of mindfulness. The Cronbach's alpha values are 0.83 in the community sample and 0.80 in the clinical sample (42).

Social support will be measured by the 12-item Multidimensional Scale of Perceived Social Support (MSPSS) (43). The scale uses a 7-point Likert scale and consists of three sources of support: family, friends and significant other. The Cronbach's alpha of the three subscales ranges from 0.85 to 0.91. (44).

Satisfaction with support group will be measured on a 7-item scale during the post-intervention. Participants will report their satisfaction with specific components and quality of service (44).

Moderators (control variables)

Positive caregiving appraisal will be measured by the 11-item Positive Aspect of Caregiving (PAC) (45). The two domains, namely, enriching life and affirming self, are measured on a 5-point Likert scale. The Cronbach's alpha is 0.89.

Caregiving self-efficacy will be measured by the 15-item Revised Scale of Caregiving Self-Efficacy (46). The scale measures caregiving self-efficacy in three domains: obtaining respite, managing disruptive patient behaviour and controlling upsetting thoughts. Participants will be asked to rate their confidence level on a continuous scale from 0 to 100% for each item. A high score indicates high confidence in carrying out caregiving tasks. The Cronbach's alpha for the three subscale ranges from 0.89 to 0.90 (47).

Health behaviour will be measured by the 6-items of self-designed statements that describe the frequency of engaging in health behaviour in the past one month by using a 4-point Likert scale. A high score indicates a high level of frequency in engaging health behaviour.

Monitoring

Since this is a low-risk intervention, which aims to support caregivers' wellness via psychosocial and behavioral techniques, no data monitoring committee is needed. There are no specific criteria for discontinuing or modifying assigned interventions, and there will be no interim analyses or predefined stopping guidelines. Participants have the freedom to discontinue their involvement in the study at any time and for any reason, as outlined in the informed consent documents. Serious adverse events and other harms from the intervention are not anticipated as this is a low-risk intervention to promote caregivers' wellness. If participants experience any potential adverse events (such as anxiety, depressed mood, etc) related to their participation, they will be reminded to promptly inform the research team. Adverse event, if applicable, will be promptly reported to the trial supervisor (KL Chou) and to the ethics committee. The trial supervisor and ethics committee will review the adverse event to determine if any action, including terminating the trial, is necessary. An independent ethics committee, separate from the sponsor, will receive an annual report on the trial's conduct, while the sponsor will review an annual progress report on the trial's progress.

Treatment fidelity

As shown in Table 1, two to six components will be delivered to participants, and the selected components in each experimental condition will be integrated seamlessly. The intervention will be administered through telephone/video-conferencing software (i.e., Zoom) by well-trained research assistants. Research assistants will be provided with 10 hours of intensive training, which will include reading materials, structured role play and practice opportunities for each component. Under experimental condition 1, in which participants will receive six components, the intervention will consist of 16 weekly sessions of approximately 45 minutes each as well as three booster support group sessions that will take place at one, two and four months after the completion of the intervention. The intervention will be completed in six weeks under experimental conditions 8, 12, 14, 15 and 16, in which participants will receive only two components.

To ensure treatment fidelity, interventionists will submit an audiotape of their first implementation of a component session for review and receive feedback from the research team. We will monitor and maintain intervention implementation through weekly supervision meetings and monthly conference calls, which will involve all interventionists. Interventionists will also submit taped intervention sessions throughout the study, and the research team will review at least 20% of the recordings. In addition, a delivery assessment form will be completed after each contact with a caregiver, and the research team will review the forms to ensure adherence to the intervention protocol.

Furthermore, interventionists will use the intervention fidelity form to record the number and duration of home visits, telephone calls and sessions. Interventionists will also rate caregivers' enactment in four aspects: data collection, home assignments, use of notebooks and use of written prescriptions (48). A satisfaction questionnaire will be conducted after the intervention to collect information about participants' satisfaction with specific components and with the quality of services (44).

Data collection procedures

After screening, interested and eligible caregivers will be scheduled for baseline assessment. They will be randomly assigned to one of the 16 experimental conditions (ECs) that vary the delivery of treatment components. Component 1 (Dementia and Caregiver Education) is compulsory and will be given to all participants as the core intervention (Table 1). Participants will be assessed at baseline (T0), 6 months of follow up (T1) and 12 months of follow-up (T2) to objectively examine the immediate and long-term effects of the multicomponent intervention. All assessments will be conducted by well-trained part-time interviewers under close supervision. Once the caregivers have completed the entire intervention, they will receive monetary compensation (HKD 400) for their participation in the study.

Sample size estimation

In the fractional factorial MOST design, the sample size to detect main effects relies on the smallest clinically important difference between the presence and absence of a component, instead of the number of components evaluated. In view of this fact and previous research, an effect size of 0.60 (Cohen's d) at 6 months is sufficient for a quasi-experimental pre‒post treatment without control to determine the sample size (41). A smaller effect size of 0.40 is selected to adopt a conservative approach. According to the power analysis, a sample size of 200 participants will give the study a power of 80% to detect the main effect or interaction effect size of 0.40 at an alpha of 0.5 with a two-tailed hypothesis test. Recruiting 250 participants will be sufficient assuming that the attrition rate is 20% over a 12-month period (41).

Data analysis

This study will adopt the principle of intention-to-treat for data analysis of the individual intervention components (components 2–6). The main objective is to examine the level of significance of each factor on proximal and primary outcomes by using linear mixed models at three time points (T0, T1 and T2). Linear mixed regression models account for repeated measures within participants and handled missing data using maximum likelihood estimation in a longitudinal dataset without needing multiple imputations.(49) The pre‒post within group difference will also be investigated (i.e., T1 vs. T0 and T2 vs. T0). On the one hand, the across time difference of each component can be viewed as the main effect. On the other hand, such effect will be modelled as components by time interactions, with T2 outcome as the primary endpoint. The study will also include the analysis for two-way interactions between components (i.e., factor 3 by factor 6 by time interaction).

The examination of moderation and mediation effects of significance will follow the guidelines established by MacKinnon and Luecken (50). To detect the underlying mechanism of components, the study will test for fit of the mediation model. The relationship path between each component and their respective primary outcomes will be established. The pathway between the components and proximal outcomes and between the proximal and primary outcomes will be investigated. The relationship between the components and primary outcomes mediated by the proximal outcomes will be investigated. When testing the fit of moderation model, the relationship between each component and the change of primary outcome will be examined. Afterwards, the strength and direction of the relationship will be tested again accounting for the effects of the baseline primary outcomes. The moderation models will be used to identify the interaction effects of the matching risk profiles of caregivers with specific component. The moderating effects of the matching profiles with components have never been explored in previous studies.

Ethical considerations and dissemination

The research has been approved by the Institutional Review Board of the Education University of Hong Kong, Human Research Ethics Committee (IRB number: A2020-2021-0045). This study is registered retrospectively in the WHO Primary Registry – Chinese Clinical Trials Registry (ChiCTR2300071235). Protocol amendments, if any, will be submitted to the ethics committee for review. Protocol amendments will only be applied after obtaining ethics approval. This trial does not involve collecting biological specimens for storage. The study aims, intervention content, voluntary participation and right to withdraw at any time will be explained verbally and outlined in detail on the information sheet. The researcher will emphasise that their decision to join/refuse/withdraw from the study will not affect the routine care and services provided by the community centres/outpatient clinics. Each participant will be given a subject code, and no identifiable information will be presented in the data file to protect participants' confidentiality. All the data collected will be stored in a secure place and can only be accessed by the research team members. There is no anticipated harm and compensation for trial participation. The trial will be conducted in accordance with the Declaration of Helsinki and reported in accordance with the CONSORT guideline and its extension to non-pharmacological interventions (51).

Results will be disseminated via presentations at scientific conferences, peer-reviewed publications, public engagement events, stakeholder organisations, patient support groups and other forms of media where appropriate. The investigators will be involved in reviewing drafts of the manuscripts, abstracts, press releases and any other publications arising from the study. The study protocol will be published in open access journal so that the public can access to the full protocol. All authors read and approved the final manuscript.

DISCUSSION

As the dementia tsunami approaches, effective family caregiver interventions are essential to improve the health outcomes of the caregiver–care recipient dyads and delay nursing home placement. Multicomponent interventions are promising strategies to support dementia caregiving but will provide a bundled treatment package, which may be associated with increased cost if inactive components are provided or if more than what is necessary is delivered. By using the MOST framework, we can identify the individually effective components of multicomponent interventions for dementia caregiving. This proposed study extends and clarifies our prior work (8-10, 13, 15, 52), indicating the efficacy of a multicomponent package of interventions to support the physical and psychosocial well-being of dementia caregivers.

We have described the protocol for a MOST fractional factorial trial aimed to optimise the multicomponent interventions for dementia caregivers. The primary objective of this study is to estimate the specific effect of each of the five components on its corresponding primary outcomes within 6 months and propose the most effective and efficient combination of components for dementia caregivers. The study has several secondary objectives, including the exploration of implementation fidelity, the main effects and the interactions of each component and the matching between treatments and individual characteristics to support dementia caregivers over the course of 12 months. The proposed trial is significant in representing the first principled and systematic effort to design an effective, efficient and potentially personalised intervention to meet the modest demands of dementia caregivers, such that all of its components (education, training on self-care skills, behavioural symptom management, behavioural activation, mindfulness and support group) are active and feasible for real-world implementation.

Limitations

The two main challenges we anticipate are related to the complexity of subject allocation and the pragmatic nature of the study. Following the MOST framework with a fractional factorial design, we are randomising participants to one of 16 conditions. Therefore, preventing contamination across conditions and monitoring the implementation fidelity of each condition are challenging.

Contamination may arise if a participant learns what other components a fellow participant is receiving, which might be most likely to occur in the study waiting areas and in the support group component when participants from different ECs come together for sharing (53). Participants may feel disappointed or disgruntled by their treatment in the study relative to other participants, which may lead to ‘resentful demoralisation’ and potentially reduced motivation to engage in the study. In addition, participants may be triggered to pursue similar types of activities or seek additional information outside of the study to compensate for what is not being received. Although eliminating contamination fully is infeasible, we will adopt several strategies to minimise it. Firstly, participants will be informed at enrolment that the treatment conditions will be varied among participants to manage expectations. The information provided in the informed consent form will follow the principle of equipoise by declaring uncertainty about the superiority of the treatment effect of all experimental conditions (54). We will also ask all the participants not to disclose their group status nor discuss any specifics of study components with other participants at any time point. For the support group session, the facilitator will attend to and encourage discussion among the four proposed themes; any discussion of other components by participants in the groups will be discouraged (55).

The second challenge relates to the fact that this study is a pragmatic trial embedded within usual care from multiple sources of recruitment, and we may lose the ability to tightly control the use of each condition. For example, if a participant chooses not to follow the use of behavioural activation strategies, we will not be able to enforce their usage. Although this may make assessment of outcome data difficult (i.e. effectiveness of a condition that is insufficiently used), this situation reflects how the conditions may be used in the 'real world'. To address this concern, we will monitor the implementation fidelity of each component. Interventionists will rate caregivers' enactment in four aspects: data collection, home assignments, use of notebooks and use of written prescriptions (56). In addition, a satisfaction questionnaire will be conducted postintervention to collect information about participants' satisfaction with specific components and with the quality of services (54).

CONCLUSION

This study uses the MOST framework to optimise the provision and delivery of a multicomponent intervention to support family dementia caregivers. The examination of the implementation fidelity, the main effects and interactions of individual components and the underlying moderating mechanism of multicomponent interventions will be useful for future investigations to optimise family caregiver interventions for implementation and dissemination. The study findings will help develop cost-effective, personalised prescription of caregiving support programs, which in turn, will enhance caregivers' physical health and psychological well-being, improve the provision of social support, help them to tackle issues of stress, anxiety and depressive symptoms and ease their burdens in caring for people with dementia, thereby reducing the costs of health care and long-term care.

Trial status

This study was retrospectively registered in the WHO Primary Registry – Chinese Clinical Trials Registry (ChiCTR2300071235). (Protocol date 30/10/2020; version identifier 2020-2021-0045). Registered 9 May, 2023. https://www.chictr.org.cn/showprojEN.html?proj=194600 This is an on-going trial (as date of protocol manuscript submission). Participants were recruited from Jun 1, 2021 to Dec 31, 2022; and the last participant follow-up visit would be on Nov 30, 2023.

DECLARATIONS

Acknowledgement

The authors would like to thank the Health and Medical Research Fund, Research Fund Secretariat, Health Bureau Hong Kong (Tel: 3150 8951, Email: rfs@healthbureau.gov.hk) for providing funding support, as well as all of the participants for their support and participation to this study. We are also grateful to the research assistants, for their dedicated assistance and contribution to this paper.

Authors Contribution

KLC had full access to all the data in the study and takes responsibility for the integrity of the data and the accuracy of the data analysis.

Study conceptualization and design: KLC (lead), JYYK (supporting), KSLC (supporting), BHPL (supporting), SZ (supporting), VWL (supporting), STC (supporting), DC (supporting), DGT (supporting)

Acquisition, analysis, or interpretation of data: KLC (lead), JYYK (supporting), KSLC (supporting), BHPL (supporting), SZ (supporting), VWL (supporting), STC (supporting), DC (supporting), DGT (supporting)

Drafting of the manuscript: Jojo KWOK (lead)

Critical revision of the manuscript for important intellectual content: KLC (lead), JYYK (supporting), KSLC (supporting), BHPL (supporting), SZ (supporting), VWL (supporting), STC (supporting), DC (supporting), DGT (supporting)

Statistical analysis: KLC (lead)

Obtained funding: KLC (lead), JYYK (supporting), KSLC (supporting), BHPL (supporting), SZ (supporting), VWL (supporting), STC (supporting), DC (supporting), DGT (supporting)

Administrative, technical, or material support: KLC (lead), JYYK (supporting), KSLC (supporting), BHPL (supporting), SZ (supporting), VWL (supporting), STC (supporting), DC (supporting), DGT (supporting)

Study supervision: KLC (lead)

Funding Statement

This study is supported by the Health and Medical Research Fund, Research Fund Secretariat, Health Bureau Hong Kong (Project number: 18191781). The funding body is not involved in the design of the study and collection, analysis, and interpretation of data and in writing the manuscript.

Availability of data and material

The data that support the findings of this study are available from K.L. Chou upon reasonable request.

Ethics approval and consent to participate

The research has been approved by the Institutional Review Board of the Education University of Hong Kong, Human Research Ethics Committee (IRB number: A2020-2021-0045). Written informed consent to participate will be obtained from all participants.

Consent for publication: Not applicable - no identifying images or other personal or clinical details of participants are presented here or will be presented in reports of the trial results. The participant information materials and informed consent form are available from the corresponding author on request.

Competing interests

The authors declare that they have no competing interests

REFERENCES

1. World Health Organization. Fact sheets of dementia Geneva, Switzerland: World Health Organization; 2021 [2023 May 22]. Available from: https://www.who.int/news-room/fact-sheets/detail/dementia.
2. Pinquart M, Sörensen S. Differences between caregivers and noncaregivers in psychological health and physical health: a meta-analysis. Psychol Aging. 2003;18(2):250-67.
3. Gaugler JE, Wall MM, Kane RL, Menk JS, Sarsour K, Johnston JA, et al. Does caregiver burden mediate the effects of behavioral disturbances on nursing home admission? Am J Geriatr Psychiatry. 2011;19(6):497-506.
4. Cheng S-T, Li K-K, Losada A, Zhang F, Au A, Thompson LW, et al. The Effectiveness of Nonpharmacological Interventions for Informal Dementia Caregivers: An Updated Systematic Review and Meta-Analysis. Psychology and aging. 2020;35(1):55-77.
5. Pinquart M, Sörensen S. Gender differences in caregiver stressors, social resources, and health: an updated meta-analysis. J Gerontol B Psychol Sci Soc Sci. 2006;61(1):P33-45.
6. Collins LM, Murphy SA, Nair VN, Strecher VJ. A strategy for optimizing and evaluating behavioral interventions. Ann Behav Med. 2005;30(1):65-73.
7. Collins LM, Kugler KC. Optimization of behavioral, biobehavioral, and biomedical interventions. Cham: Springer International Publishing. 2018;10(1007):978-3.
8. Belle SH, Burgio L, Burns R, Coon D, Czaja SJ, Gallagher-Thompson D, et al. Enhancing the quality of life of dementia caregivers from different ethnic or racial groups: a randomized, controlled trial. Annals of internal medicine. 2006;145(10):727-38.
9. Au A, Li S, Lee K, Leung P, Pan PC, Thompson L, et al. The Coping with Caregiving Group Program for Chinese caregivers of patients with Alzheimer's disease in Hong Kong. Patient education and counseling. 2010;78(2):256-60.
10. Cheung KS, Lau BH, Wong PW, Leung AY, Lou VW, Chan GM, et al. Multicomponent intervention on enhancing dementia caregiver well-being and reducing behavioral problems among Hong Kong Chinese: a translational study based on REACH II. Int J Geriatr Psychiatry. 2015;30(5):460-9.
11. Penrod J, Baney B, Loeb SJ, McGhan G, Shipley PZ. The influence of the culture of care on informal caregivers' experiences. ANS Adv Nurs Sci. 2012;35(1):64-76.
12. Whitebird RR, Kreitzer M, Crain AL, Lewis BA, Hanson LR, Enstad CJ. Mindfulness-based stress reduction for family caregivers: a randomized controlled trial. The Gerontologist. 2013;53(4):676-86.
13. Kwok JYY, Kwan JCY, Auyeung M, Mok VCT, Lau CKY, Choi KC, et al. Effects of Mindfulness Yoga vs Stretching and Resistance Training Exercises on Anxiety and Depression for People With Parkinson Disease: A Randomized Clinical Trial. JAMA Neurology. 2019;76(7):755-63.
14. Kwok JYY, Choi EPH, Lee JJ, Lok KYW, Kwan JCY, Mok VCT, et al. Effects of Mindfulness Yoga Versus Conventional Physical Exercises on Symptom Experiences and Health-related Quality of Life in People with Parkinson’s Disease: The Potential Mediating Roles of Anxiety and Depression. Annals of Behavioral Medicine. 2022;56(10):1068-81.
15. Cheung DSK, Kor PPK, Jones C, Davies N, Moyle W, Chien WT, et al. The Use of Modified Mindfulness-Based Stress Reduction and Mindfulness-Based Cognitive Therapy Program for Family Caregivers of People Living with Dementia: A Feasibility Study. Asian Nurs Res (Korean Soc Nurs Sci). 2020;14(4):221-30.
16. Kor PPK, Liu JYW, Chien WT. Effects of a Modified Mindfulness-Based Cognitive Therapy for Family Caregivers of People With Dementia: A Randomized Clinical Trial. The Gerontologist. 2020;61(6):977-90.
17. Cheung DSK, Kor PPK, Jones C, Davies N, Moyle W, Chien WT, et al. The Use of Modified Mindfulness-Based Stress Reduction and Mindfulness-Based Cognitive Therapy Program for Family Caregivers of People Living with Dementia: A Feasibility Study. Asian nursing research. 2020;14(4):221-30.
18. Cheng ST, Li KK, Losada A, Zhang F, Au A, Thompson LW, et al. The effectiveness of nonpharmacological interventions for informal dementia caregivers: An updated systematic review and meta-analysis. Psychol Aging. 2020;35(1):55-77.
19. Zarit SH. Past is prologue: how to advance caregiver interventions. Aging & mental health. 2018;22(6):717-22.
20. Wu CFJ, Hamada MS. Experiments : planning, analysis, and optimization. 2 ed. New York: Wiley; 2009.
21. Gwadz MV, Collins LM, Cleland CM, Leonard NR, Wilton L, Gandhi M, et al. Using the multiphase optimization strategy (MOST) to optimize an HIV care continuum intervention for vulnerable populations: a study protocol. Bmc Public Health. 2017;17.

22. Cheng ST, Chan ACM. The Center for Epidemiologic Studies Depression Scale in older Chinese: thresholds for long and short forms. Int J Geriatr Psych. 2005;20(5):465-70.
23. Yu JH, Yap P, Liew TM. The optimal short version of the Zarit Burden Interview for dementia caregivers: diagnostic utility and externally validated cutoffs. Aging Ment Health. 2019;23(6):706-10.
24. Schulz R, Gallagher-Thompson D, Haley W, Czaja S. Understanding the intervention process: A theoretical/conceptual framework for intervention approaches to caregiving. In: Schulz R, editor. Handbook on dementia caregiving: evidence-based interventions in family caregivers. New York: Springer; 2000. p. P.33-60.
25. Kwok T, Wong B, Ip I, Chui K, Young D, Ho F. Telephone-delivered psychoeducational intervention for Hong Kong Chinese dementia caregivers: a single-blinded randomized controlled trial. Clin Interv Aging. 2013;8:1191-7.
26. Au A, Li S, Lee K, Leung P, Pan PC, Thompson L, et al. The Coping with Caregiving Group Program for Chinese caregivers of patients with Alzheimer's disease in Hong Kong. Patient Educ Couns. 2010;78(2):256-60.
27. Gallagher-Thompson D, Gray HL, Tang PCY, Pu CY, Leung LYL, Wang PC, et al. Impact of in-home behavioral management versus telephone support to reduce depressive symptoms and perceived stress in Chinese caregivers: Results of a pilot study. Am J Geriat Psychiat. 2007;15(5):425-34.
28. Wang LQ, Chien WT. Randomised controlled trial of a family-led mutual support programme for people with dementia. J Clin Nurs. 2011;20(15-16):2362-6.
29. Lam CLK, Tse EYY, Gandek B. Is the standard SF-12 Health Survey valid and equivalent for a Chinese population? Qual Life Res. 2005;14(2):539-47.
30. Fong DYT, Wong JYH, Choi EPH, Lam KF, Kwok C. The English and Chinese language versions of the Short Form 12-item Health Survey are equivalent. Health and quality of life outcomes. 2021;19(1).
31. Tang JYM, Ho AHY, Luo H, Wong GHY, Lau BHP, Lum TYS, et al. Validating a Cantonese short version of the Zarit Burden Interview (CZBI-Short) for dementia caregivers. Aging Ment Health. 2016;20(9):996-1001.
32. Leung DYP, Lam TH, Chan SSC. Three versions of Perceived Stress Scale: validation in a sample of Chinese cardiac patients who smoke. Bmc Public Health. 2010;10.
33. Huang FF, Wang HJ, Wang ZH, Zhang JG, Du WW, Su C, et al. Psychometric properties of the perceived stress scale in a community sample of Chinese. Bmc Psychiatry. 2020;20(1).
34. Li RH. Reliability and validity of a shorter Chinese version for Ryff's psychological well-being scale. Health Educ J. 2014;73(4):446-52.
35. Shek DTL. The Chinese Version of the State-Trait Anxiety Inventory - Its Relationship to Different Measures of Psychological Well-Being. J Clin Psychol. 1993;49(3):349-58.
36. Shek DTL. Reliability and Factorial Structure of the Chinese Version of the State-Trait Anxiety Inventory. J Psychopathol Behav. 1988;10(4):303-17.
37. Boey KW. Cross-validation of a short form of the CES-D in Chinese elderly. Int J Geriatr Psychiatry. 1999;14(8):608-17.
38. Lee DTF, Thompson DR, Yu SF, Woo J. Reliability and validity of the Medical Outcomes Study Social Support Survey (Chinese version). J Am Geriatr Soc. 2005;53(5):920-1.
39. Yu, Lee, Woo. Psychometric testing of the Chinese version of the Medical Outcomes Study Social Support Survey (MOS-SSS-C). Res Nurs Health. 2004;27(2):135-43.
40. Belle SH, Burgio L, Burns R, Coon D, Czaja SJ, Gallagher-Thompson D, et al. Enhancing the quality of life of dementia caregivers from different ethnic or racial groups - A randomized, controlled trial. Annals of internal medicine. 2006;145(10):727-38.
41. Cheung, Lau, Wong, Leung, Lou, Chan, et al. Multicomponent intervention on enhancing dementia caregiver well-being and reducing behavioral problems among Hong Kong Chinese: a translational study based on REACH II. Int J Geriatr Psych. 2015;30(5):460-9.
42. Chien WT. Testing the psychometric properties of a Chinese version of Dementia Management Strategies Scale. Neuropsych Dis Treat. 2015;11:1663-75.
43. Deng YQ, Li S, Tang YY, Zhu LH, Ryan R, Brown K. Psychometric Properties of the Chinese Translation of the Mindful Attention Awareness Scale (MAAS). Mindfulness. 2012;3(1):10-4.
44. Burgio, Collins, Schmid, Wharton, McCallum, DeCoster. Translating the REACH Caregiver Intervention for Use by Area Agency on Aging Personnel: the REACH OUT Program. Gerontologist. 2009;49(1):103-16.
45. Lou VWQ, Lau BHP, Cheung KSL. Positive aspects of caregiving (PAC): Scale validation among Chinese dementia caregivers (CG). Arch Gerontol Geriat. 2015;60(2):299-306.

46. Au A, Lai MK, Lau KM, Pan PC, Lam L, Thompson L, et al. Social support and well-being in dementia family caregivers: the mediating role of self-efficacy. Aging Ment Health. 2009;13(5):761-8.
47. Au, Lau, Sit, Cheung, Lai, Wong, et al. The Role of Self-Efficacy in the Alzheimer's Family Caregiver Stress Process: A Partial Mediator between Physical Health and Depressive Symptoms. Clin Gerontologist. 2010;33(4):298-315.
48. Burgio, Stevens, Guy, Roth, Haley. Impact of two psychosocial interventions on white and African American family caregivers of individuals with dementia. Gerontologist. 2003;43(4):568-79.
49. Twisk J, de Boer M, de Vente W, Heymans M. Multiple imputation of missing values was not necessary before performing a longitudinal mixed-model analysis. Journal of clinical epidemiology. 2013;66(9):1022-8.
50. MacKinnon DP, Luecken LJ. How and for whom? Mediation and moderation in health psychology. Health Psychol. 2008;27(2s):S99-s100.
51. Schulz KF, Altman DG, Moher D, Group C. CONSORT 2010 statement: updated guidelines for reporting parallel group randomised trials. BMJ. 2010;340:c332.
52. Kwok JYY, Choi EPH, Wong JYH, Lok KYW, Ho MH, Fong DYT, et al. A randomized clinical trial of mindfulness meditation versus exercise in Parkinson's disease during social unrest. NPJ Parkinson's disease. 2023;9(1):7.
53. Broder-Fingert S, Kuhn J, Sheldrick RC, Chu A, Fortuna L, Jordan M, et al. Using the Multiphase Optimization Strategy (MOST) framework to test intervention delivery strategies: a study protocol. Trials. 2019;20(1):728.
54. Kwok JYY, Kwan JCY, Auyeung M, Mok VCT, Chan HYL. The effects of yoga versus stretching and resistance training exercises on psychological distress for people with mild-to-moderate Parkinson’s disease: Study protocol for a randomized controlled trial. Trials. 2017;18(1):1-13.
55. Wang LQ, Chien WT. Randomised controlled trial of a family-led mutual support programme for people with dementia. J Clin Nurs. 2011;20(15-16):2362-6.
56. Burgio LD, Collins IB, Schmid B, Wharton T, McCallum D, Decoster J. Translating the REACH caregiver intervention for use by area agency on aging personnel: the REACH OUT program. The Gerontologist. 2009;49(1):103-16.
