# Supplementary material for: Multicomponent Intervention for Distressed Informal Caregivers of People With Dementia: A Randomized Clinical Trial
Source: JAMA Netw Open. 2025 Mar 17;8(3):e250069. doi: 10.1001/jamanetworkopen.2025.0069 (PMC11915064; doi:10.1001/jamanetworkopen.2025.0069)
Supplement: Supplement 2. — eTable 1. Experimental Conditions of the Fractional Factorial Randomized Clinical Trial eFigure 1. Supposed Impact of the Different Intervention Components on the Stress–Health Process of the Informal Caregiver eFigure 2. Mechanism of the Multicomponent Intervention eTable 2. Satisfaction Level of Family Dementia Caregivers by Each Component (N = 250) eTable 3. Analysis of Main Effects of Primary Outcomes Corresponding to Each Tested Component Using the Mixed Models at Baseline, 6 Months of Follow-Up, and 12 Months of Follow-Up eTable 4. Analysis of Main Effects of Proximal Outcomes Using the Mixed Models at Baseline, 6 Months of Follow-Up, and 12 Months of Follow-Up [file jamanetwopen-e250069-s002.pdf]

Supplemental Online Content

Kwok JYY, Cheung DSK, Zarit S, et al. Multicomponent intervention for distressed informal caregivers of people with dementia: a randomized clinical trial. *JAMA Netw Open*. 2025;8(3):e250069. doi:10.1001/jamanetworkopen.2025.0069

eTable 1. Experimental Conditions of the Fractional Factorial Randomized Clinical Trial

eFigure 1. Supposed Impact of the Different Intervention Components on the Stress–Health Process of the Informal Caregiver

eFigure 2. Mechanism of the Multicomponent Intervention

eTable 2. Satisfaction Level of Family Dementia Caregivers by Each Component (N = 250)

eTable 3. Analysis of Main Effects of Primary Outcomes Corresponding to Each Tested Component Using the Mixed Models at Baseline, 6 Months of Follow-Up, and 12 Months of Follow-Up

eTable 4. Analysis of Main Effects of Proximal Outcomes Using the Mixed Models at Baseline, 6 Months of Follow-Up, and 12 Months of Follow-Up

This supplemental material has been provided by the authors to give readers additional information about their work.

© 2025 Kwok JYY et al. *JAMA Network Open*.

eTable 1. Experimental Conditions of the Fractional Factorial Randomized Clinical Trial

Experimental conditions	n	Intervention components						Total n
		Psychoeducation (n=250)	Self-care skill training (n=127)	Behavioral symptom management (n=125)	Behavioral activation (n=126)	Mindfulness-based intervention (n=122)	Social support group (n=122)	
1	16	Yes	Yes	Yes	Yes	Yes	Yes	96
2	16	Yes	Yes	Yes	Yes	No	No	64
3	16	Yes	Yes	Yes	No	Yes	No	64
4	17	Yes	Yes	Yes	No	No	Yes	68
5	16	Yes	Yes	No	Yes	Yes	No	64
6	15	Yes	Yes	No	Yes	No	Yes	60
7	15	Yes	Yes	No	No	Yes	Yes	60
8	16	Yes	Yes	No	No	No	No	32
9	16	Yes	No	Yes	Yes	Yes	No	64
10	16	Yes	No	Yes	Yes	No	Yes	64
11	12	Yes	No	Yes	No	Yes	Yes	48
12	16	Yes	No	Yes	No	No	No	32
13	15	Yes	No	No	Yes	Yes	Yes	60
14	16	Yes	No	No	Yes	No	No	32
15	16	Yes	No	No	No	Yes	No	32
16	16	Yes	No	No	No	No	Yes	32

© 2025 Kwok JYY et al. *JAMA Network Open*.

eFigure 1. Supposed Impact of the Different Intervention Components on the Stress–Health Process of the Informal Caregiver

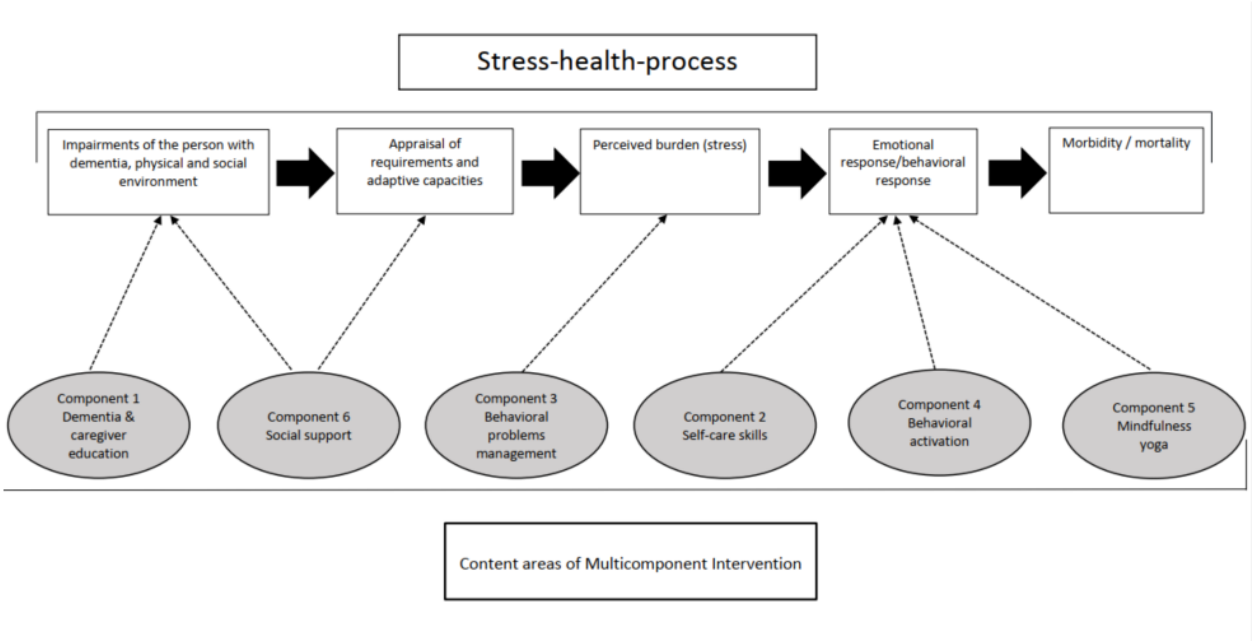

Adapted from Schulz, 2000.

© 2025 Kwok JYY et al. *JAMA Network Open*.

eFigure 2. Mechanism of the Multicomponent Intervention

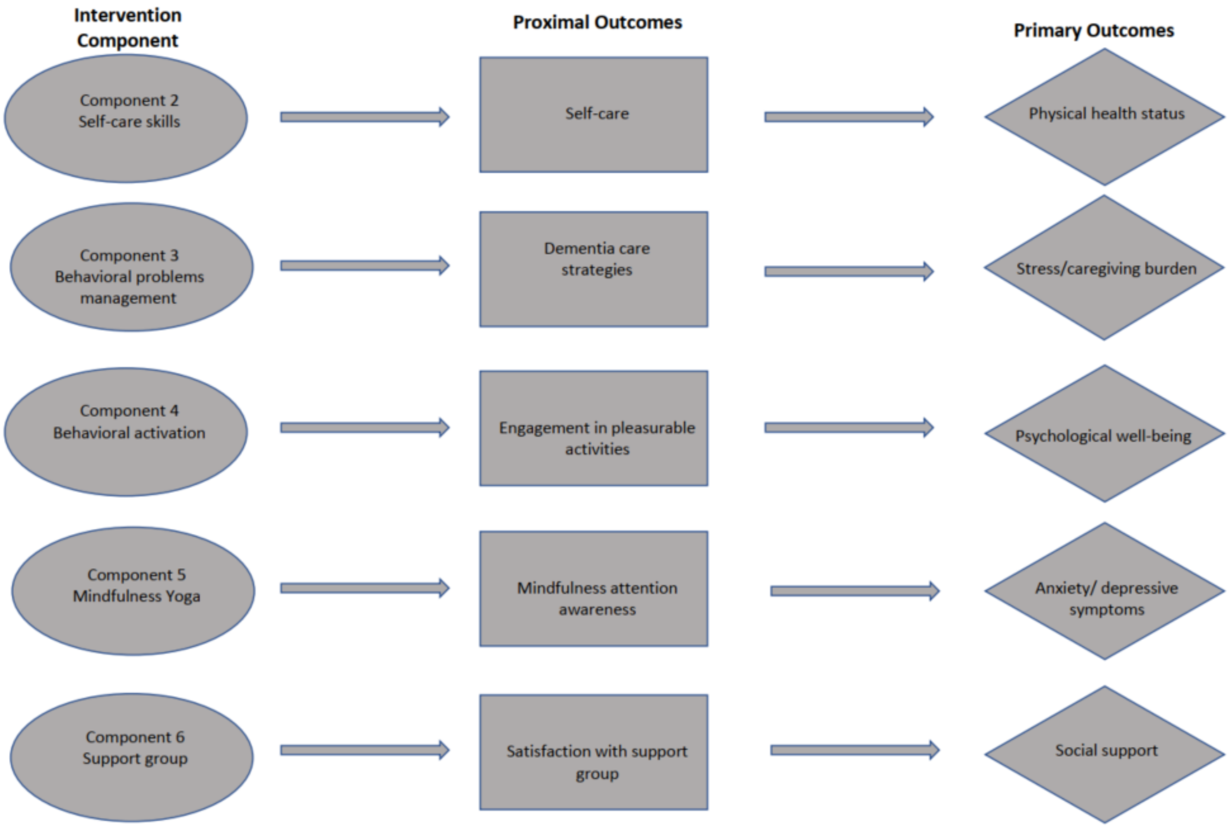

© 2025 Kwok JYY et al. *JAMA Network Open*.

eTable 2. Satisfaction Level of Family Dementia Caregivers by Each Component (N = 250)

		Total n 250	6-mo Follow-up			12-mo Follow-up		
			Mean	SD	*p*-value	Mean	SD	*p*-value
**Self-care skills (SC)**	Presence	127	27.4	3.6	.084	26.7	3.5	.058
	Absence	123	26.7	3.2		25.9	3.2	
**Behavioral problem management (BPM)**	Presence	125	27.1	2.8	.988	26.1	3.3	.554
	Absence	125	27.1	3.9		26.4	3.5	
**Behavioral activation (BA)**	Presence	126	27.0	3.7	.663	26.1	3.6	.430
	Absence	124	27.2	3.2		26.4	3.2	
**Mindfulness-based intervention (MBI)**	Presence	122	26.5	3.4	.005	26.8	3.3	.027
	Absence	128	27.7	3.4		25.8	3.4	
**Support group (SG)**	Presence	122	27.0	3.4	.671	26.0	3.2	.144
	Absence	128	27.2	3.5		26.6	3.5	

Note: All participants analyzed according to allocation (n=250).
Satisfaction with the support group – a measure of satisfaction, range 5-35, higher scores indicate greater level of satisfaction.
The change in scores from 12 months to 6 months was compiled to assess the degree of change in satisfaction over time.

© 2025 Kwok JYY et al. *JAMA Network Open*.

eTable 3. Analysis of Main Effects of Primary Outcomes Corresponding to Each Tested Component Using the Mixed Models at Baseline, 6 Months of Follow-Up, and 12 Months of Follow-Up

				Baseline	Estimated marginal means (95% CI)		Estimated difference of scores (Presence - Absence) Mean difference (95% CI)				Effect size for change scores	Overall between-group difference *p*-value
			N		6-mo Follow-up	12-mo Follow-up	6-mo Follow-up	*p*-value	12-mo Follow-up	*p*-value		
**Physical Health Status**												
**SF-12 (Physical)**	**SCS**	Pre	127	50.10	49.64 (48.24 ,51.03)	49.29 (47.84, 50.74)	1.30 (-0.07, 2.68)	0.062	1.18 (-0.24, 2.61)	0.104	0.15 (-0.73, 1.03)	0.732
		Abs	123	50.97	48.33 (46.90 ,49.77)	48.11 (46.61, 49.60)						
**Caregiver Burden and General Stress**												
**ZBI**	**BPM**	Pre	125	20.66	18.66 (17.07 ,20.24)	17.25 (15.71, 18.79)	0.94 (-0.64, 2.52)	0.243	0.54 (-1, 2.08)	0.489	-0.27 (-1.15, 0.61)	0.540
		Abs	125	20.25	17.72 (16.17 ,19.27)	16.71 (15.21, 18.22)						
**PSS**	**BPM**	Pre	125	18.63	17.06 (16.03 ,18.10)	16.27 (15.31, 17.24)	-0.29 (-1.32, 0.74)	0.576	-0.40 (-1.36, 0.56)	0.415	-0.03 (-0.66, 0.59)	0.915
		Abs	125	18.74	17.35 (16.34 ,18.36)	16.67 (15.73, 17.61)						
**Psychological Well-being**												
**PWB**	**BA**	Pre	126	66.67	67.96 (65.53 ,70.39)	68.28 (66.13, 70.43)	0.29 (-2.10, 2.69)	0.809	1.16 (-0.96, 3.28)	0.282	0.64 (-0.56, 1.84)	0.294
		Abs	124	65.88	67.67 (65.35 ,69.99)	67.12 (65.06, 69.17)						
**Anxiety and Depressive Symptoms**												
**HADS-A**	**MBI**	Pre	122	5.89	4.99 (4.12 ,5.87)	4.66 (3.82, 5.49)	0.23 (-0.65, 1.12)	0.629	0.02 (-0.82, 0.87)	0.960	-0.36 (-0.8, 0.08)	0.106
		Abs	128	5.73	4.76 (3.89 ,5.63)	4.63 (3.80, 5.46)						
**PHQ-9**	**MBI**	Pre	122	13.34	12.60 (11.92 ,13.27)	10.91 (10.22, 11.60)	1.37(0.69, 2.06)	<0.001	0.68(-0.02, 1.38)	0.056	-1.11 (-1.4, -0.83)	<0.001
		Abs	128	12.41	11.22 (10.55 ,11.90)	10.23 (9.54, 10.91)						
**Perceived Adequacy of Functional Social Support**												
**MOS-SSS**	**SG**	Pre	122	64.93	65.59 (62.34 ,68.84)	66.37 (63.44, 69.3)	2.32 (-1.08, 5.73)	0.182	3.58 * (0.51, 6.65)	0.022	2.32 (0.63, 4.01)	0.007
		Abs	128	63.40	63.27 (59.64 ,66.89)	62.79 (59.52, 66.06)						

© 2025 Kwok JYY et al. *JAMA Network Open*.

Abbreviations:

- Components: SCS: Self-care skills, BPM: Behavioral problems management, BA: Behavioral activation, MBI: Mindfulness-based intervention, and SG: Support group.
- SF-12 (Physical), 12-item Short-Form Health Survey, range 0-100, with higher scores indicating a better physical health condition.
- ZBI, Zarit Burden Interview scale, range 0-48, with higher scores indicating greater caregiver burden.
- PSS, Perceived Stress Scale, range 0-40, with higher scores indicating higher levels of stress.
- PWB, Ryff's Psychological Wellbeing Scale, range 16-96, with higher scores indicating better psychological wellbeing.
- HADS-A, Anxiety subscale of Hospital Anxiety and Depression Scale, range 0-21, with higher scores indicating higher levels of anxiety.
- PHQ-9, Patient Health Questionnaire-9, range 0-27, with higher scores indicating greater severity of depressive symptoms.
- MOS-SSS, Medical Outcomes Study – Social Support Survey, range 19-95, with higher scores indicating higher level of perceived social support.

*** $p < .001$, ** $p < .01$, * $p < .05$

© 2025 Kwok JYY et al. *JAMA Network Open*.

eTable 4. Analysis of Main Effects of Proximal Outcomes Using the Mixed Models at Baseline, 6 Months of Follow-Up, and 12 Months of Follow-Up

				Baseline	Estimated marginal means (95% CI)		Estimated difference of scores (Presence - Absence) Mean difference (95% CI)				Effect size for change scores	Overall between-group difference *p*-value
			N		6-mo Follow-up	12 mo Follow-up	6-mo Follow-up	*p*-value	12 mo Follow-up	*p*-value		
**Physical Health Status**												
**RAM-SC**	**SCS**	Pre	127	3.80	4.98 (4.43 ,5.53)	5.15 (4.59, 5.71)	0.78 (0.24, 1.32)	0.005	0.81 (0.26, 1.36)	0.004	0.25 (-0.09, 0.59)	0.142
		Abs	123	3.77	4.21 (3.64 ,4.77)	4.34 (3.76, 4.91)						
**Caregiver Burden and General Stress**												
**DMSS Criticism**	**BPM**	Pre	125	28.72	27.17 (25.68, 28.66)	27.73 (26.4, 29.06)	1.63(0.14, 3.12)	0.032	1.94 (0.61, 3.26)	0.004	0.47 (-0.43, 1.37)	0.304
		Abs	125	27.26	25.54 (24.09, 27.00)	25.8 (24.5, 27.09)						
**DMSS Encouragement**	**BPM**	Pre	125	22.77	21.82 (20.66, 22.98)	21.96 (20.9, 23.02)	-0.33 (-1.48, 0.83)	0.576	0.45 (-0.61, 1.51)	0.400	1.06 (0.21, 1.91)	0.015
		Abs	125	23.06	22.15 (21.02, 23.28)	21.51 (20.47, 22.54)						
**DMSS Active Management**	**BPM**	Pre	125	35.84	34.52 (33.10, 35.93)	34.56 (33.34, 35.77)	-1.10 (-2.52, 0.31)	0.125	0.09 (-1.12, 1.31)	0.883	1.77 (0.77, 2.77)	0.001
		Abs	125	36.86	35.62 (34.24, 37.00)	34.47 (33.28, 35.65)						
**Psychological Well-being**												
**No. of meaningful Events**	**BA**	Pre	127	1.21	0.99 (0.79 ,1.19)	0.86 (0.66, 1.06)	-0.24 (-0.44, -0.04)	0.017	-0.27 (-0.46, -0.07)	0.011	-0.11 (-0.26, 0.04)	0.153
		Abs	123	1.35	1.24 (1.03 ,1.44)	1.13 (0.94, 1.32)						
**Anxiety and Depressive Symptoms**												
**FFMQ**	**MBI**	Pre	122	61.66	62.73 (60.86, 64.59)	63.03 (61.31, 64.76)	-2.36 (-4.24, -0.47)	0.015	-1.05 (-2.79, 0.70)	0.239	2.27 (1.28, 3.26)	<0.001
		Abs	128	64.15	65.08 (63.23, 66.94)	64.08 (62.36, 65.79)						
**Satisfaction with support group**												
**Satisfaction**	**SG**	Pre	122	-	-	27.2 (26.19, 28.21)	-	-	-0.73 (-1.55, 0.1)	0.083	-0.44(-1.39, 0.51)	0.364
		Abs	128	-	-	27.93 (26.71, 29.15)						

© 2025 Kwok JYY et al. *JAMA Network Open*.

Abbreviations:

- Components: SCS: Self-care skills, BPM: Behavioral problems management, BA: Behavioral activation, MBI: Mindfulness-based intervention, and SG: Support group.
- RAM-SC, Risk Appraisal Measure – Self-care, range 0-11, with higher scores indicating a lower perceived risk.
- DMSS, Dementia Management Strategies Scale, categorized into three subscales of caregiving strategies: criticism, encouragement and active management, with higher scores indicating more frequent corresponding caregiving behaviors.
- No. of meaningful events, range 0-5, higher numbers indicate more meaningful events in the past week.
- FFMQ, Five Facet Mindfulness Questionnaire, a measure of mindfulness with regards to thoughts, experiences, and actions in daily life, range 20-100, higher scores indicate higher levels of mindfulness.
- Satisfaction, a measure of satisfaction with the support group, range 5-35, higher scores indicate greater level of satisfaction. The change in scores from 12 months to 6 months was compiled to assess the degree of change in satisfaction over time.

*** $p < .001$, ** $p < .01$, * $p < .05$

© 2025 Kwok JYY et al. *JAMA Network Open*.
