# Supplementary material for: Multicomponent Intervention for Distressed Informal Caregivers of People With Dementia: A Randomized Clinical Trial
Source: JAMA Netw Open. 2025 Mar 17;8(3):e250069. doi: 10.1001/jamanetworkopen.2025.0069 (PMC11915064; doi:10.1001/jamanetworkopen.2025.0069)
Supplement: Supplement 3. — Data Sharing Statement [file jamanetwopen-e250069-s003.pdf]

Data Sharing Statement

Kwok. Multicomponent Intervention for Distressed Informal Caregivers of People With Dementia. *JAMA Netw Open*. Published March 17, 2025.
doi:10.1001/jamanetworkopen.2025.0069

Data
Additional Information: WHO Primary Registry – Chinese Clinical Trials Registry: ChiCTR2300071235 https://www.chictr.org.cn/showprojEN.html?proj=194600
Data available: No

Additional Information
Explanation for why data not available: The data supporting the findings of this study are not publicly available due to the presence of personal information that could potentially compromise the privacy of the research participants but are available from KL Chou upon reasonable request.
